# Isoform-specific characterization of class I histone deacetylases and their therapeutic modulation in pulmonary hypertension

**DOI:** 10.1038/s41598-020-69737-x

**Published:** 2020-07-30

**Authors:** Prakash Chelladurai, Swati Dabral, Sobha Rani Basineni, Chien-Nien Chen, Mario Schmoranzer, Nina Bender, Christine Feld, René Reiner Nötzold, Gergana Dobreva, Jochen Wilhelm, Benno Jungblut, Lan Zhao, Uta-Maria Bauer, Werner Seeger, Soni Savai Pullamsetti

**Affiliations:** 1 Max-Planck Institute for Heart and Lung Research, Bad Nauheim, Germany; 2 German Center for Lung Research (DZL), Giessen, Germany; 3 Center for Pharmacology and Therapeutics, Experimental Medicine, Hammersmith Hospital, Imperial College London, London, UK; 4 Institute of Molecular Biology and Tumor Research, Philipps University Marburg, Marburg, Germany; 5 Department of Anatomy and Developmental Biology, CBTM, Medical Faculty Mannheim, Heidelberg University, Mannheim, Germany; 6 Department of Internal Medicine, Justus-Liebig-University Giessen, Klinikstrasse 36, 35392 Giessen, Germany

**Keywords:** Histone post-translational modifications, Transcriptomics, RNAi, Gene expression profiling, Respiratory tract diseases, Diseases

## Abstract

Pharmacological modulation of class I histone deacetylases (HDAC) has been evaluated as a therapeutic strategy for pulmonary hypertension (PH) in experimental models of PH. However, information of their expression, regulation and transcriptional targets in human PH and the therapeutic potential of isoform-selective enzyme modulation are lacking. Comprehensive analysis of expression and regulation of class I HDACs (HDAC1, HDAC2, HDAC3 and HDAC8) was performed in cardiopulmonary tissues and adventitial fibroblasts isolated from pulmonary arteries (PAAF) of idiopathic pulmonary arterial hypertension (IPAH) patients and healthy donors. Cellular functions and transcriptional targets of HDAC enzymes were investigated. Therapeutic effects of pan-HDAC (Vorinostat), class-selective (VPA) and isoform-selective (CAY10398, Romidepsin, PCI34051) HDAC inhibitors were evaluated ex vivo (IPAH-PAAF, IPAH-PASMC) and in vivo (rat chronic hypoxia-induced PH and zebrafish angiogenesis). Our screening identifies dysregulation of class I HDAC isoforms in IPAH. Particularly, HDAC1 and HDAC8 were consistently increased in IPAH-PAs and IPAH-PAAFs, whereas HDAC2 and HDAC8 showed predominant localization with ACTA2-expressing cells in extensively remodeled IPAH-PAs. Hypoxia not only significantly modulated protein levels of deacetylase (HDAC8), but also significantly caused dynamic changes in the global histone lysine acetylation levels (H3K4ac, H3K9/K14ac and H3K27ac). Importantly, isoform-specific RNA-interference revealed that HDAC isoforms regulate distinct subset of transcriptome in IPAH-PAAFs. Reduced transcript levels of KLF2 in IPAH-PAAFs was augmented by HDAC8 siRNA and HDAC inhibitors, which also attenuated IPAH-associated hyperproliferation and apoptosis-resistance ex vivo*,* and mitigated chronic hypoxia-induced established PH in vivo, at variable degree. Class I HDAC isoforms are significantly dysregulated in human PAH. Isoform-selective HDAC inhibition is a viable approach to circumvent off-target effects.

## Introduction

Pulmonary arterial hypertension (PAH) is a cardiopulmonary disorder, characterized by a progressive increase in pulmonary artery pressure, usually culminating in right ventricular (RV) failure^1^. In addition to genetic predisposition, increasing evidence suggests a significant involvement of epigenetic changes in the pathogenesis of PAH^2,3^. Histone post-translational modifications (PTM) such as acetylation of lysine residues on the N-terminal tails of histones modulate chromatin structure and accessibility by altering DNA-histone interactions^4^. Histone acetyltransferases (HAT) are a class of enzymes that catalyze the transfer of acetyl group from acetyl-CoA to the lysine ε-amino groups on the N-terminal tails of histones^5^. Whereas, histone deacetylases (HDAC) contain a highly conserved deacetylase domain and are responsible for the removal of acetyl groups^6^. Among the two enzymes families regulating histone acetylation, HDACs are extensively studied in cardiovascular diseases^7^. Of the eighteen mammalian HDAC isoforms (or isoenzymes) identified, class I HDACs (HDAC1-3, and 8) are Zn^2+^-dependent deacetylases and differ in structure, enzymatic function, subcellular localization, and expression patterns^8^.

Interestingly, the contribution of aberrant HDAC activity to the pathogenesis of PH and RV hypertrophy is postulated majorly based on the promising therapeutic benefits of small-molecule HDAC inhibitors observed in different animal models of PH. Similar to the therapeutic benefits demonstrated by HDAC inhibitors in animal models of left ventricle dysfunction^9^, the therapeutic potential of several commercially available broad-spectrum HDAC inhibitors (Vorinostat) and class I HDAC targeting (Valproic acid, Sodium butyrate, Apicidin, Mocetinostat, Entinostat) were demonstrated to successfully attenuate PH and RV hypertrophy in multiple rodent models of PH^3,10^. Even though numerous studies demonstrated therapeutic benefit upon pan-HDAC inhibition in pre-clinical PH models, the detrimental effects of trichostatin A (TSA) leading to worsened RV function in pulmonary artery banding (PAB) model questioned the safety profile of using HDAC inhibitors for patients with RV failure. The mixed results of HDAC inhibition on RV function highlights the need for identification of specific HDAC isoforms dysregulated in human PAH.

With regards to the expression profile of HDAC isoforms in PH, few studies reported elevated class I HDAC catalytic activity that correlated with increased abundance of HDAC1, HDAC2, and HDAC3 protein levels in bovine PH-fibroblasts^11^ and in lung tissue homogenates from hypoxia-induced PH rats and IPAH patients^12^. However, site-specific regulation of individual HDAC isoforms in different cardiopulmonary tissues (PA, RV) and pulmonary vascular cells in human PAH, has not been characterized yet. As well, the functional role of individual class I HDACs isoforms in driving PH is sparsely addressed. Moreover, before designing selective therapeutic targeting approaches, it is important to be acquainted with the expression pattern of individual HDAC isoforms in different tissues and vascular cells of the cardiopulmonary system in human PAH. This would enable us to circumvent the harmful side-effects of broad-spectrum HDAC inhibition. Therefore, we comprehensively characterized the expression, cellular functions, and transcriptional targets of class I HDAC isoforms in different cardiopulmonary tissues and evaluated the therapeutic effects of pan-HDAC and selective-HDAC inhibition ex vivo and in vivo*.*

## Results

### Tissue-specific dysregulation of class I HDAC isoforms in human IPAH

**Figure 1:**
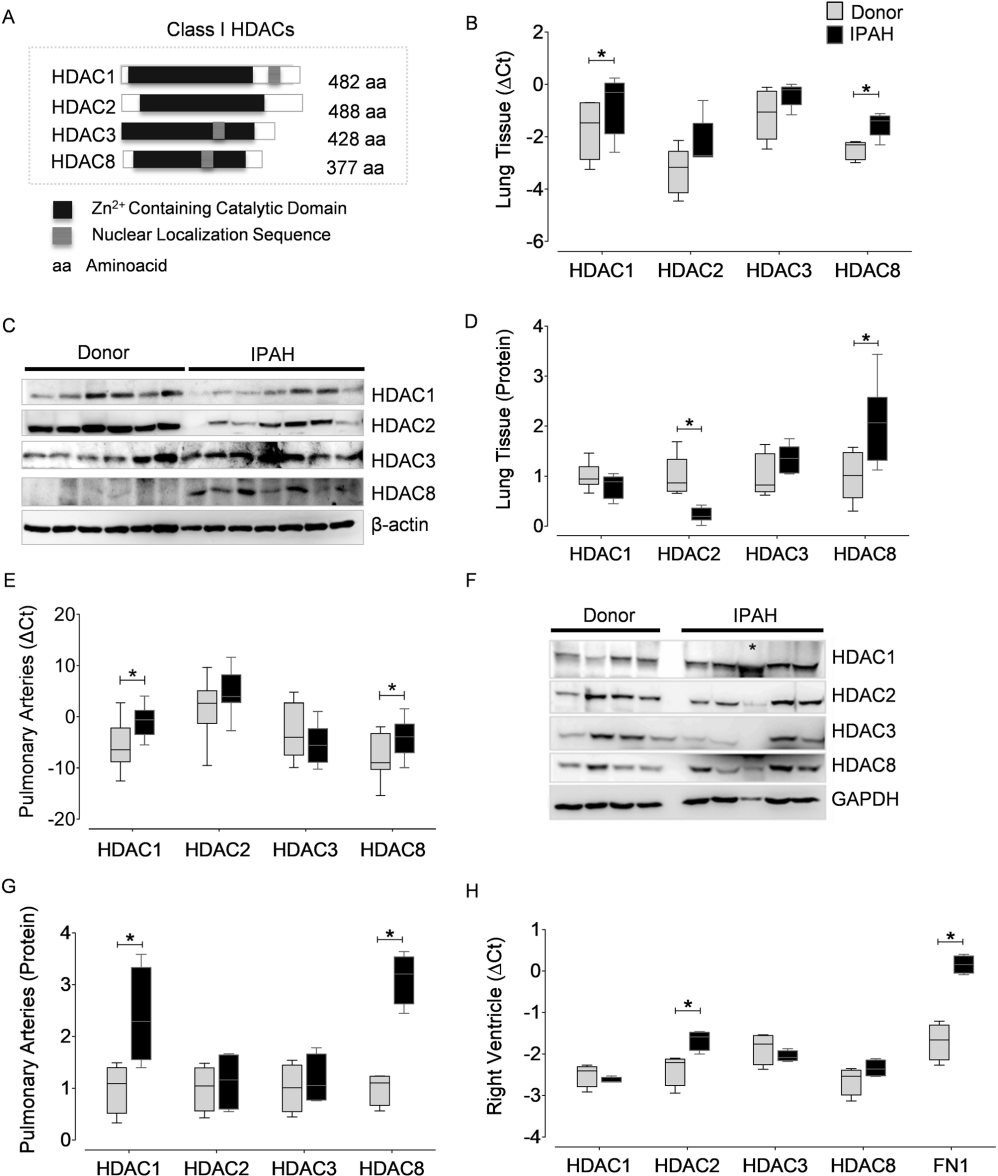
Expression of class I HDAC isoforms are altered in different cardiopulmonary tissues from human PAH. (**A**) Schematic representation of the mammalian class I HDACs. (**B**) Real-time quantitative polymerase chain reaction (qPCR) analysis was performed with primer pairs (Supplementary Table 1) on the complementary DNA (cDNA) synthesized from the RNA isolated from human lung homogenates of healthy donors (n = 5) and IPAH (n = 5) patients. All values were normalized against hypoxanthine phosphoribosyltransferase (HPRT). (**C**) Western blot was performed on human lung homogenates from donors (n = 6) and PAH (n = 7) patients using validated antibodies listed in Supplementary Table 2. (**D**) Blots from lung homogenates were quantified by densitometry and are represented as box plots after normalization to internal loading control. (**E**) The transcription levels of HDAC isoforms were quantified using qPCR on the total RNA isolated from the laser micro-dissected intrapulmonary arteries (> 50 µm and < 200 µm) dissected from the lungs of human donor (n = 12) and PAH (n = 10) patients. Results are presented as expression relative to that of HPRT using the ∆C_t_ method. (**F**) Western blots were performed on the lysates prepared from donor (n = 4) and PAH (n = 5) pulmonary arteries (PA). (**G**) Blots from PAs were further quantified by densitometry and are represented as bar charts. GAPDH was used as a loading control. Asterisk symbol (*) within the western blot indicates that the protein was isolated from the PAs of an Eisenmenger syndrome patient, and was not included in the quantitative comparison of protein expression levels between PAH patients and donors. Besides, Eisenmenger syndrome is also classified as a part of Group 1 (PAH, 1.4.4.1) in the clinical classification of PH. (**H**) qPCR analysis was performed on the RNA isolated from human right ventricle of healthy donors (n = 4) and PAH (n = 4) patients. *p < 0.05 (Student's t-test), indicates significant differences between PAH and donors.

To profile the changes in the expression pattern of class I HDAC isoforms (Fig. 1A) in IPAH, we characterized the gene and protein expression levels of HDAC1, HDAC2, HDAC3 and HDAC8 in different human tissues including heart, lung and pulmonary arteries (PA) obtained from IPAH and donor lungs. Although lung tissue comprises numerous cell types, analysis of IPAH-lungs indicated upregulation of HDAC1 and HDAC8 transcripts (Fig. 1B), while only HDAC8 protein levels were significantly elevated in IPAH (Fig. 1C, D). Furthermore, to capture the expression pattern of HDAC isoforms in PAH, distal PAs were isolated by laser-assisted microdissection from donor and IPAH lungs. Real-time PCR analysis revealed significantly elevated levels of HDAC1 and HDAC8 mRNA in IPAH-PAs compared to donors (Fig. 1E). Increased transcript levels in PAs corroborated with the significantly increased protein levels of HDAC1 and HDAC8 isoforms in protein lysates prepared from IPAH-PAs (Fig. 1F, G). In the context of PAH-associated RV dysfunction, human RV biopsies obtained from IPAH patients and donors were screened and HDAC2 transcripts were found to be significantly elevated (Fig. 1H).

### Immunohistochemical analysis reveals differential distribution of HDAC isoforms in remodeled pulmonary vasculature

**Figure 2:**
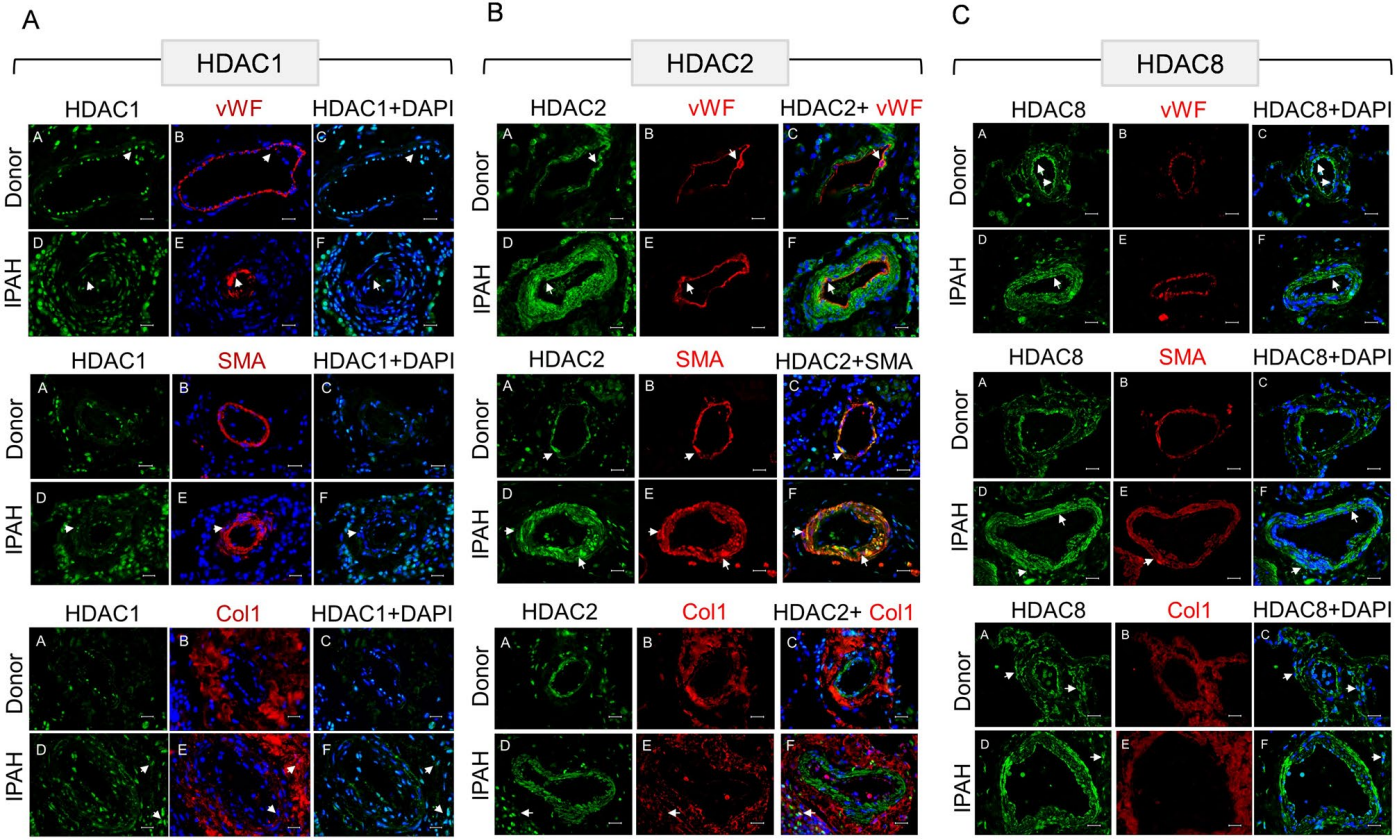
Immunolocalization of class I HDAC isoforms in human lungs from donors and IPAH patients. Representative microscopic pictures of human pulmonary arteries immunostained for (**A**) HDAC1, (**B**) HDAC2 and (**C**) HDAC8 expression in human donor and IPAH lung sections. HDAC (in green fluorescence) expression was localized to different vascular by staining for cell-specific markers such as von Willebrand factor (vWF, Red) for endothelial cells, α-smooth muscle actin (SMA or ACTA2, Red) for smooth muscle cells and collagen I (Col1 or COL1A1, Red) associated with adventitial fibroblasts, while the nucleus was counter-stained with DAPI (Blue). Scale bar: 50 µm.

To determine the in vivo distribution of HDAC isoform expression in different vascular cell compartments (intima, media and adventitia) of distal PAs, immunohistochemical analysis was performed on human donor- and IPAH- lung sections. To begin with, immunofluorescence stainings performed on adventitial fibroblasts isolated from pulmonary arteries (PAAF) ex vivo revealed predominant nuclear localization of HDAC1, HDAC2, and HDAC8 proteins (Supplementary Fig. 1A). Contrary to the confined nuclear HDAC1 expression in endothelial cells (PAEC) lining the lumen of normal PAs, the remodeled PAs in IPAH-lungs displayed strong nuclear immunoreactivity for HDAC1 protein in all resident vascular cell types, including PAECs, smooth muscle cells (PASMC) and PAAFs (Fig. 2A). Specifically, the number of cells immunostained for HDAC1 protein was strikingly increased in the adventitial layer of IPAH-PAs, which may prominently include PAAFs and immune infiltrating cells. Remarkably, strong immunoreactivity for HDAC2 protein was detected throughout the remodeled medial SMC layers and the adventitial compartment of IPAH-PAs (Fig. 2B). With regards to the distribution of HDAC8 in donor-PAs, a regimented expression of HDAC8 protein was observed in the endothelial cells lining the non-remodeled PAs, besides the adventitial compartment (Fig. 2C). Contrary to the predominant endothelial expression of HDAC8 in donor-PAs, immunolocalization of HDAC8 in IPAH-PAs confirmed increased immunoreactivity in the medial SMC layers of the remodeled vessels, as well as in the intima and adventitia of these distal PAs.

### Class I HDAC isoforms are overexpressed in IPAH and are associated with fibroblast proliferation

**Figure 3:**
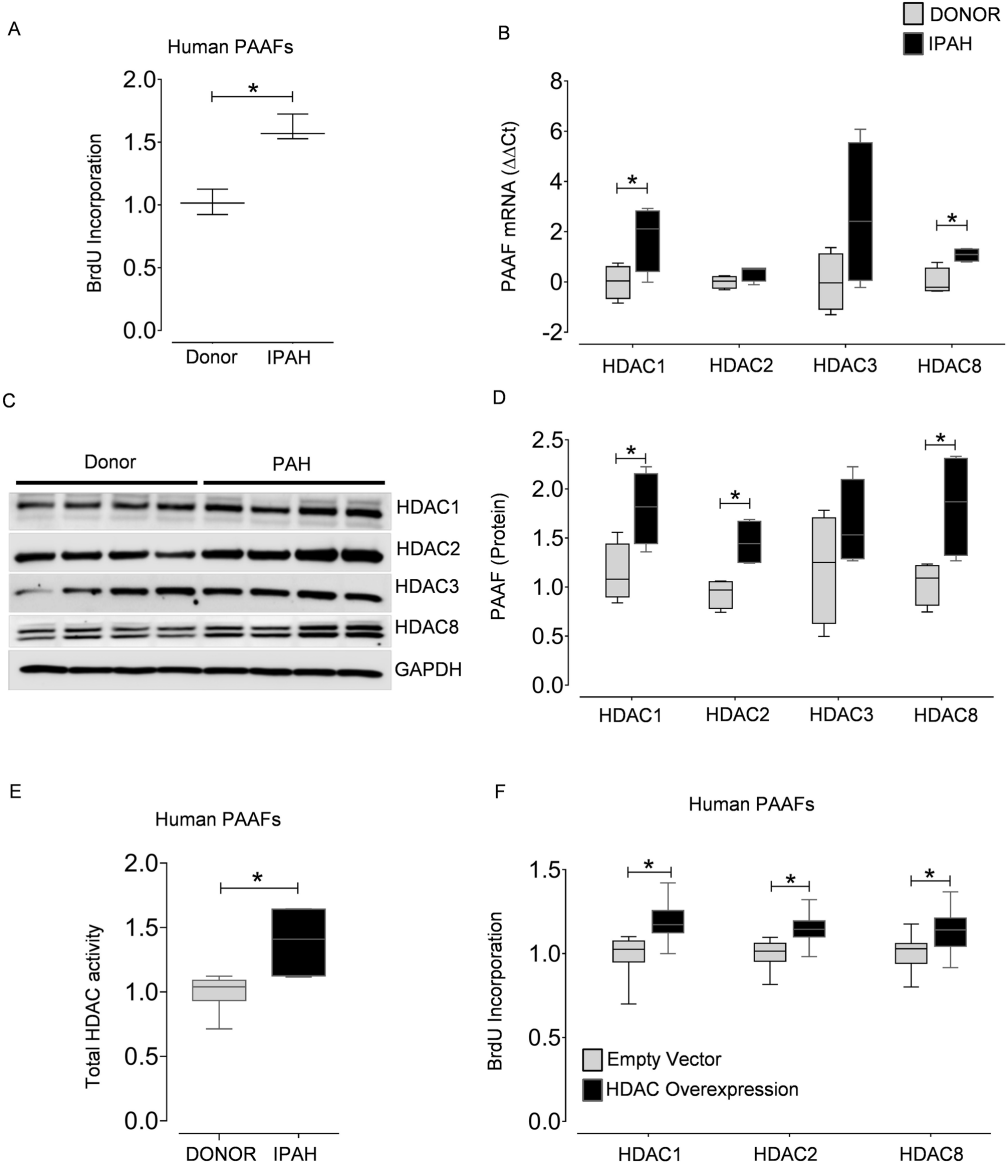
Class I HDAC isoforms are overexpressed in PAH and are associated with adventitial fibroblast proliferation. Cell proliferation was assessed by BrdU incorporation in (**A**) donor- and IPAH-PAAFs (n = 3) cultured ex vivo. Data was normalized to respective donors. (**B**) qPCR analysis of HDACs was performed on the RNA isolated from the PAAFs harvested from healthy donors (n = 4) and PAH (n = 4) patients. ∆C_t_ values were normalized to β-2 microglobulin (β2M) and further normalized (∆∆C_t_) to donor controls. (**C**) Western blots were performed on lysates extracted from human PAAFs of healthy donors (n = 4) and PAH (n = 4) patients. (**D**) Blots were quantified by densitometry and are represented as box plots after normalization to internal loading control. (**E**) HDAC activity (OD/min/mg) was measured in PAAFs harvested from healthy donors (n = 3) and PAH (n = 3) patients using colorimetric assay kit (Epigenase HDAC Activity/Inhibition Assay Kit, Epigentek). (**F**) Overexpression of HDACs was achieved by transient transfection of donor-PAAFs (n = 3) with validated HDAC1, HDAC2, HDAC8 plasmids, and their functional effects on proliferation was assessed by BrdU incorporation, 48 hours (h) post-transfection. Empty vector plasmid was used as negative control. Data was normalized to untransfected cells. In all plots, significant differences between corresponding comparisons are indicated as *p < 0.05, Student's t-test.

**Figure 4:**
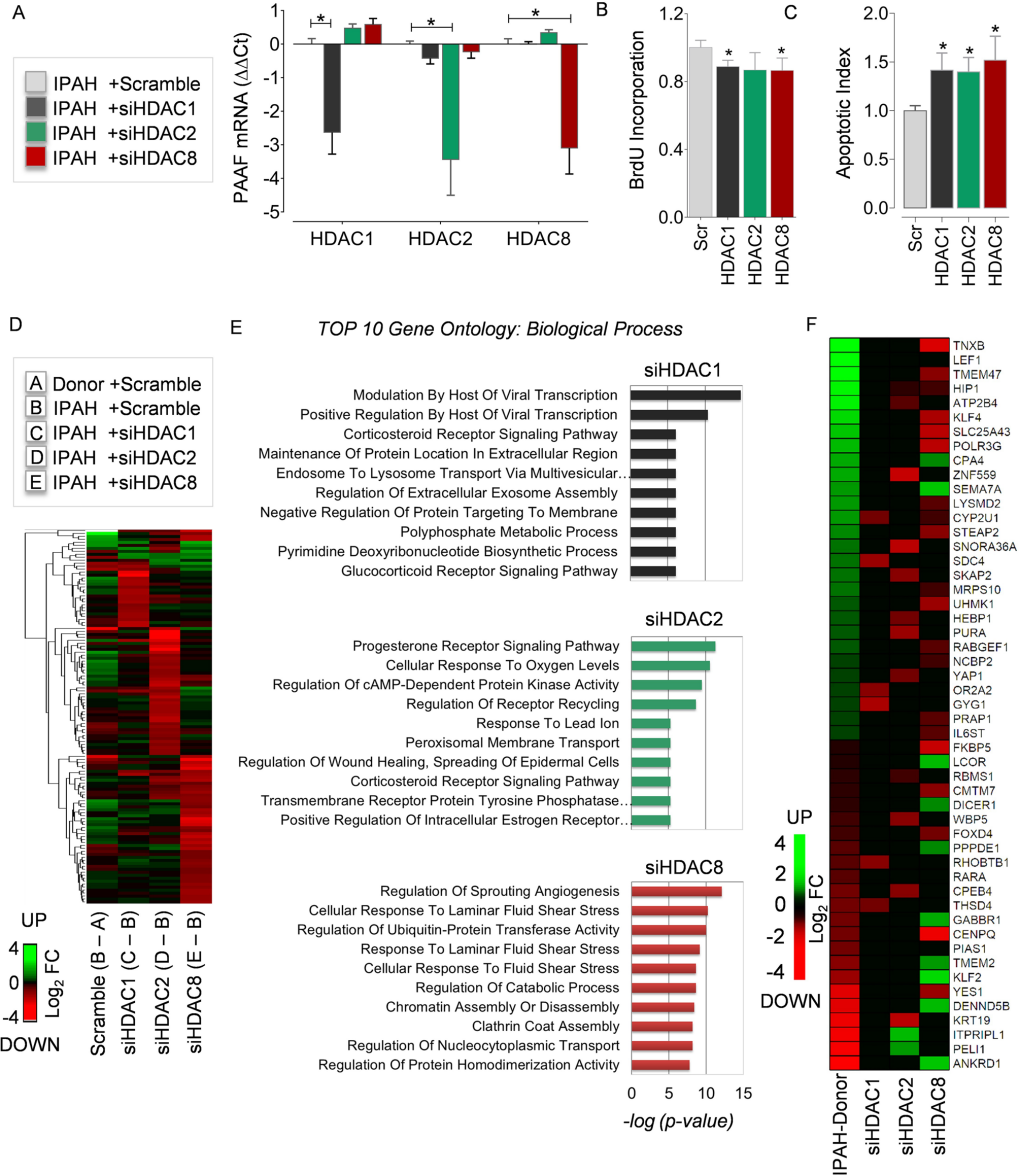
Transcriptome and pathways regulated by HDAC isoforms in PAH. RNA-interference was achieved by transient transfection of IPAH-PAAFs cultured ex vivo with validated HDAC1, HDAC2 and HDAC8 siRNAs. (**A**) qPCR analysis was performed on the RNA isolated from IPAH-PAAFs (n = 3), 24 h post-transfection. ∆C_t_ values were calculated using β2M as reference and further normalized (∆∆C_t_) to the respective solvent concentrations (DMSO or water). (**B**) Functional effects of RNA-interference on fetal calf serum (FCS) induced IPAH-PAAF proliferation was assessed by BrdU incorporation, 48 h post-transfection. Similarly, (**C**) functional effects of RNA-interference on serum starvation (0.1% FCS) induced apoptosis in IPAH-PAAFs was assessed by Cell Death Detection ELISA^PLUS^, 48 h post-transfection. Data was normalized to untransfected cells and visualized (IPAH; n = 3; *p < 0.05 versus scrambled siRNA, Student's t-test). (**D**) Transcriptomic alterations between IPAH and donor-PAAFs (B-A) and the genome-wide transcriptional targets of individual HDAC isoforms was profiled using microarray-based expression analysis following RNA-interference in IPAH-PAAFs, along with scrambled siRNA as a negative control. Heatmap shows hierarchical clustering of all genes selected for at least one of the comparisons C-B, D-B, and E-B (log_2_FC ≤ − 1 or log_2_FC ≥  + 1, FDR < 0.05) (Supplementary Table 3). The colors indicate log_2_ fold-differences between the compared groups. Green and red indicate higher and lower expression, respectively, in the group that is given first, compared to the group given second. (**E**) Gene ontology (GO) enrichment analysis was performed to identify GO terms associated with biological process (BP, − log_2_(*p-value*) < 0.05) and top 10 GO terms for each comparison (C-B, D-B, and E-B) were displayed as bar charts (Supplementary Table 4). (**F**) Differentially expressed protein coding genes that are differentially expressed in comparison (B-A) and in at least one of the siRNA knockdown comparisons (C-B, D-B, E-B) were enlisted and displayed as a heatmap (Supplementary Table 3). *FC* Fold change, *FDR* False discovery rate.

Before profiling the expression of HDAC isoforms in vascular cells, we characterized the phenotypic differences exhibited by vascular cells isolated ex vivo from PAs of IPAH and donor lungs. In accordance with the previous reports^3^, we observed a significant increase in BrdU incorporation in IPAH-PAAFs compared to donors (Fig. 3A). Even though, only the transcript levels of HDAC1 and HDAC8 were elevated in IPAH-PAAFs (Fig. 3B), the protein expression levels of HDAC1, HDAC2, HDAC8, and total HDAC activity were significantly increased in IPAH-PAAFs compared to donors (Fig. 3C-E). Since the expression levels of HDAC isoforms are significantly elevated in IPAH-PAAFs, we investigated their functional roles by exogenous expression of HDAC1, HDAC2, and HDAC8 isoforms in donor-PAAFs, which promoted PAAF proliferation (Fig. 3F, Supplementary Fig. 1B,C). Conversely, RNA-interference of all three HDAC isoforms (Fig. 4A, Supplementary Fig. 5A) decreased hyperproliferative phenotype of IPAH-PAAFs (Fig. 4B, Supplementary Fig. 5B) or IPAH-PASMCs (Supplementary Fig. 5C) and increased apoptosis in IPAH-PAAFs (Fig. 4C, Supplementary Fig. 2A,B) at different degrees.

**Figure 5:**
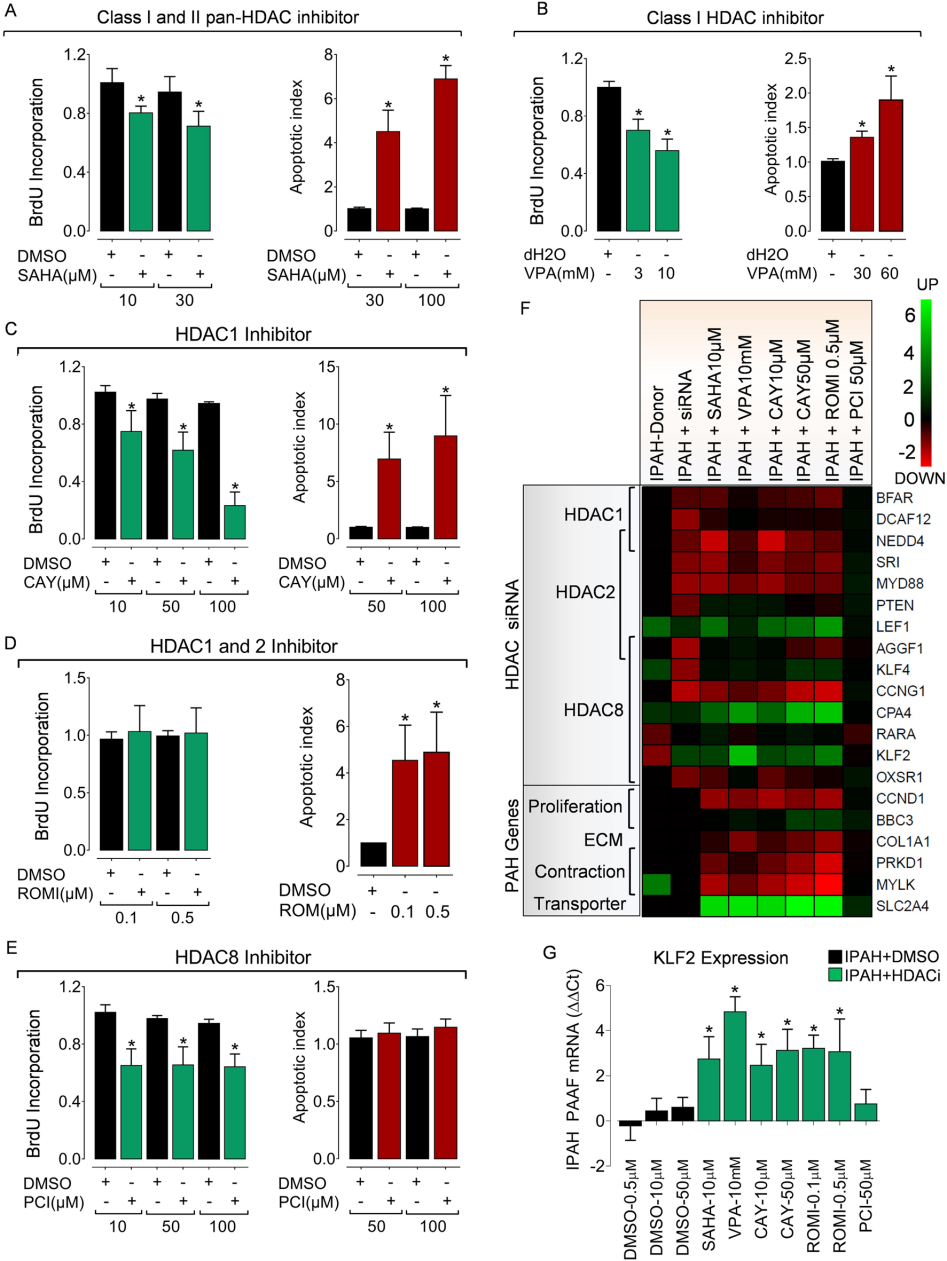
Isoform-selective HDAC activity inhibition reverses hypertensive phenotypes in PAH fibroblasts ex vivo. Pharmacological HDAC inhibition suppresses hyper-proliferative phenotype and reverses resistance to apoptosis in IPAH-PAAFs ex vivo. IPAH-PAAFs were treated with increasing concentrations of commercially available (**A**) pan-HDAC inhibitor Vorinostat (SAHA), (**B**) class-selective Valproic acid (VPA) and isoform selective inhibitors such as (**C**) CAY10398, (**D**) Romidepsin, (**E**) PCI34051 or their respective solvents (DMSO or water). Cell proliferation was assessed by BrdU incorporation and induction of apoptosis was assessed by Cell Death Detection ELISA^PLUS^, 24 h post-treatment. Absorbance values obtained for HDAC inhibitor and solvent treatments were normalized to the BrdU incorporation of untreated cells. Data are represented as mean ± SEM (n = 3; *p < 0.05 versus DMSO or water, Student's t-test). (**F**) The impact of HDAC activity inhibition on the modulation of transcription targets of HDAC isoforms (Fig. 4D) and PAH-relevant genes in IPAH-PAAFs (n = 3) was evaluated by qPCR. ∆C_t_ values were calculated using β2M as reference. Inhibitor treatments were further normalized (∆∆C_t_) to the respective solvent concentrations (DMSO, dd.H2O). Heatmap representation also includes Log_2_ fold change values (Supplementary Table 3) obtained from the microarray dataset (columns 1 and 2). (**G**) Example plot visualizing relative KLF2 mRNA (∆∆C_t_) expression. Data are represented as mean ± SEM (n = 3; *p < 0.05 versus solvent control, Student's t-test).

### Transcriptional targets of HDAC isoforms in IPAH

Furthermore, to identify the genome-wide transcriptional targets of HDAC1, HDAC2, and HDAC8 isoforms in PAH, RNA-interference was performed in IPAH-PAAFs. Global transcriptome analysis revealed statistically significant differential expression of 2,210 genes with at least two fold differential expression (|Log_2_FC| ≥ 1, FDR ≤ 0.05) between IPAH-PAAFs and donor-PAAFs (Lane B-A; Fig. 4D and Supplementary Fig. 2C). Notably, RNA interference of HDAC1 (Lane C-B), HDAC2 (Lane D-B), and HDAC8 (Lane E-B) isoforms in PAH-PAAFs, specifically modulated transcription of a subset of genes (Fig. 4D) and signaling pathways (Fig. 4E), in comparison to the scrambled siRNA treated (controls) IPAH-PAAFs. Hierarchical clustering of differentially expressed genes in IPAH-PAAFs following RNA-interference visualizes distinct subset of genes regulated by HDAC1 (48 genes), HDAC2 (86 genes) and HDAC8 (127 genes) isoforms (Fig. 4D). HDACs are typically considered as transcriptional co-repressors that induce local condensation of chromatin (Wang et al.^13^). Interestingly, the transcripts differentially expressed upon knockdown of all three HDACs were mostly downregulated. This corroborates with the previous observation that the majority of HDACs in the human genome are associated with active genes and only a minor fraction is detected in silent genes^13,14^.

One of the important genes upregulated in IPAH and downregulated upon HDAC2 knockdown was transcriptional regulator yes-associated protein 1 (YAP1) (Fig. 4F, Supplementary Fig. 3I), which is typically linked to the development of stiffness-dependent remodeling and fibrotic phenotypes in both idiopathic pulmonary fibrosis and pulmonary vascular disease^15^. Another transcription factor differentially expressed in IPAH and regulated by HDAC2 is lymphoid enhancer-binding factor-1 (LEF1). LEF1 is a downstream nuclear effector of Wnt/β-catenin signaling pathway, but can also modulate gene transcription independently and is associated with epithelial-mesenchymal transition^16^.

In this study, we found LEF1 transcripts and protein were significantly upregulated in hyperproliferative IPAH-PAAFs compared to donors (Supplementary Fig. 3A–C). This correlated with the significant in vivo distribution of LEF1 protein immunoreactivity in severely remodeled IPAH-PAs than donors (Supplementary Fig. 3D). With regards to the regulation of LEF1 promoter by HDAC2, chromatin immunoprecipitation of HDAC1 and HDAC2 confirmed enriched HDAC2 occupancy at LEF1 promoter in IPAH, compared to donors (Supplementary Fig. 3E). Whereas, IPAH-PAAFs subjected to either HDAC2-specific RNA-interference (Supplementary Fig. 3F) or HDAC inhibitor treatments (Fig. 5F) further increased the transcription of LEF1. Moreover, overexpression of HDAC2, but not HDAC1 upregulates transcription of LEF1 mRNA in donor-PAAFs (Supplementary Fig. 3G). Overexpression of LEF1 increased proliferation of normal fibroblasts (Supplementary Fig. 3H,I). Overall, LEF1 is indeed increased in IPAH-PAs and regulated by HDAC2 in IPAH-PAAFs and promotes fibroblast proliferation.

The transcriptional targets and associated biological process regulated upon downregulation of HDAC8 are regulation of sprouting angiogenesis and laminar fluid shear stress (Fig. 4D). Reduced krüppel-like factor 2 (KLF2) signaling is implicated in PAH pathogenesis^17^. Remarkably, our data demonstrate that laminar shear stress-induced transcription factor KLF2 is not only transcriptionally repressed in IPAH (Fig. 4), but can be restored to baseline levels upon isoform-specific downregulation of HDAC8 expression or HDAC activity inhibition (Figs. 4E, 5G).

### Isoform-selective HDAC activity inhibition reverses hypertensive phenotypes in IPAH fibroblasts ex vivo

Acquired hyperproliferative capacity and resistance to apoptosis are the major pathological hallmarks exhibited by vascular cells during vascular remodeling^3^. Before the in vivo evaluation of selective HDAC inhibition in a pre-clinical rodent model of PH, IPAH-PAAFs were subjected to treatment with pan-HDAC inhibitor SAHA and class-selective VPA, isoform-selective HDAC inhibitors CAY10398 (HDAC1), Romidepsin (HDAC1/ HDAC2) and PCI34051 (HDAC8). Both pan-HDAC inhibitor SAHA and class-selective VPA dose-dependently caused a significant reduction in BrdU incorporation and induced apoptosis in IPAH-PAAFs (Fig. 5A–C, E), and IPAH-PASMCs (Supplementary Fig. 4A, B). Notably, isoform-selective inhibitors (CAY10398, PCI34051) significantly attenuated the hyper-proliferative phenotype of IPAH-PAAFs (Fig. 5C, E). However, no significant effect on BrdU incorporation was observed with romidepsin (Fig. 5D). Similar to the levels of apoptosis induced by pan-HDAC inhibition, significant induction of apoptosis was observed with isoform-selective HDAC inhibitors CAY10398 and Romidepsin, but not with PCI34051. Overall, under ex vivo conditions, both pan-HDAC and isoform-selective HDAC inhibitors exerted comparable anti-proliferative and pro-apoptotic effects in IPAH vascular cells, thus confirming their promising potential to significantly reverse hypertensive phenotypes exhibited by PAH vascular cells.

We further assessed the impact of HDAC inhibition on the transcriptional targets of HDAC isoforms (Fig. 5E) and found that both pan- and selective- HDAC inhibitors parallel the regulatory effects of isoform-specific RNA-interference for a subset of genes (Fig. 5F). Additionally, HDAC inhibition also reversed the expression of multiple genes that were previously implicated in PAH phenotypes. Notably, HDAC inhibition downregulated genes associated with proliferation (CCND1), fibrosis (COL1A1) and smooth muscle contractility (MYLK), but upregulated the expression of pro-apoptotic BBC3, glucose transporter member 4 (SLC2A4) (Fig. 5F), and flow-responsive KLF2 (Fig. 5F, G) in IPAH-PAAFs.

### Expression and activity of HDAC isoforms are altered under hypoxia

**Figure 6:**
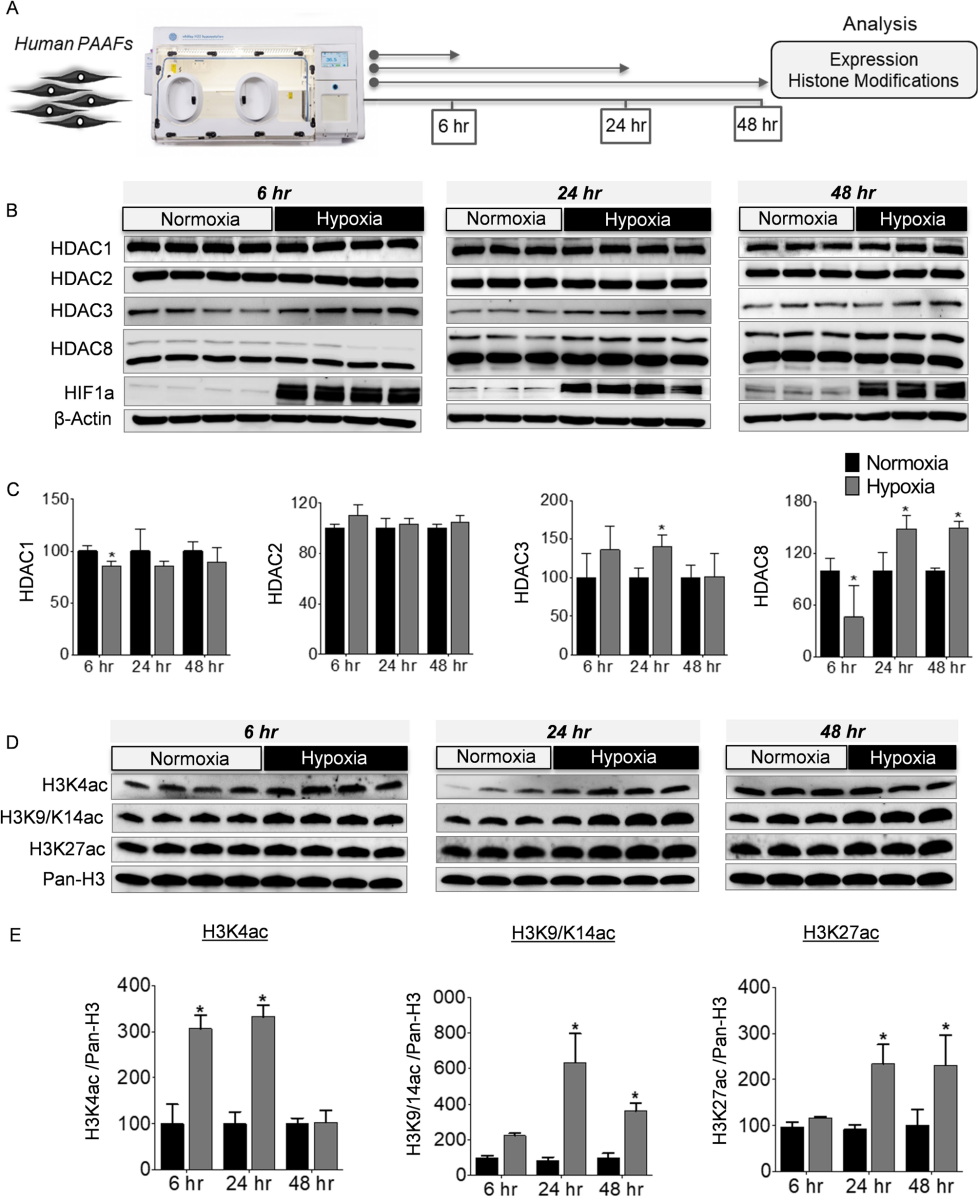
Regulation of class I HDACs and histone modifications by hypoxia ex vivo. (**A**) Schematic representation of the experimental plan to study the regulation of HDAC expression and dynamic changes in histone acetylation levels in donor-PAAFs (n ≥ 3) exposed to hypoxia. (**B**) Western blots were performed on proteins extracted from human donor-PAAFs exposed to hypoxia. (**C**) Blots were quantified by densitometry and are represented as bar charts after normalization to internal loading control, β-actin. For HDAC8, both the upper (Fig. 6) and lower bands (in Fig. S3) were quantified separately. (**D**) To determine the effect of hypoxia exposure on histone modifications, western blot analysis was performed on extracts from donor-PAAFs exposed to hypoxia with antibodies raised against specific post-translational modification of histones associated with transcription activation (H3K4ac; H3K9/K14ac) and active enhancers (H3K27ac). (**E**) Blots were quantified by densitometric analysis and are represented as bar charts after normalization to internal loading control (Pan-histone H3). Significant differences found in comparison between the treatment (hypoxia) and control groups (normoxia) are indicated by an asterisk symbol (*p < 0.05, Two-way ANOVA with Bonferroni post-tests for multiple comparisons). K-Lysine, *ac* acetylation.

The recruitment of HAT and HDAC enzymes to a specific chromatin locus is one of the major determinants in the transcriptional regulation of genes controlling cellular phenotypes such as proliferation and apoptosis^18^. To identify the upstream mechanisms regulating expression and activity HDAC enzymes in PAH, we exposed the normal human PAAFs to hypoxia, a known PH stimulus, for 6, 24, and 48 hours (h) (Fig. 6A). Although the protein levels of HDAC1 and HDAC2 did not show much fluctuation under hypoxia, there was a mild decrease in the protein levels of HDAC1 and HDAC8 at 6 h (Fig. 6B, C). Upon prolonged hypoxia exposure, a significant elevation of both HDAC3 and HDAC8 protein levels was observed at 24 h, while only HDAC8 protein levels were significantly elevated under hypoxia at 48 h (Fig. 6B, C). Since the aberrant expression and activity of HDAC enzymes can consequently affect histone acetylation levels, we probed for changes in global histone acetylation levels, to indirectly investigate the presence of an imbalance between the activities of HDAC or HAT enzymes that regulate acetylation levels of histones under hypoxia. We quantified the time-dependent changes in global levels of histone H3 acetylation, associated with actively transcribed promoters (H3K4ac, H3K9/K14ac) and active enhancers (H3K27ac) (Fig. 6D). Hypoxia induced a significant increase in H3K4ac at 6 and 24 h (Fig. 6D, E), which hints an early increase in the activity of HAT enzymes under hypoxia. The early increase in global histone acetylation levels may indicate adaptive chromatin remodeling driven by hypoxia exposure. However, the elevated H3K4ac levels were restored to the normoxic baseline levels by 48 h, which may be attributable to a decline in activity of HAT enzymes or elevated activity of HDAC enzymes. Similarly, H3K9/K14ac and H3K27ac levels were significantly elevated in response to hypoxia at 24 and 48 h. Contrary to the early increases in H3K4ac and H3K9/K14ac, H3K27 had a stable increase in acetylation levels 24 h after exposure to hypoxia (Fig. 6D, E). This experiment demonstrates that hypoxic stress induces a substantial imbalance in the chromatin regulatory mechanisms involving histone acetylation and deacetylation, via aberrant expression and recruitment of HDAC and HAT enzymes in human pulmonary artery fibroblasts exposed to hypoxia.

### Pan-HDAC inhibitors strongly attenuate hypoxia-induced PH and RV hypertrophy compared to isoform-selective inhibitors

**Figure 7:**
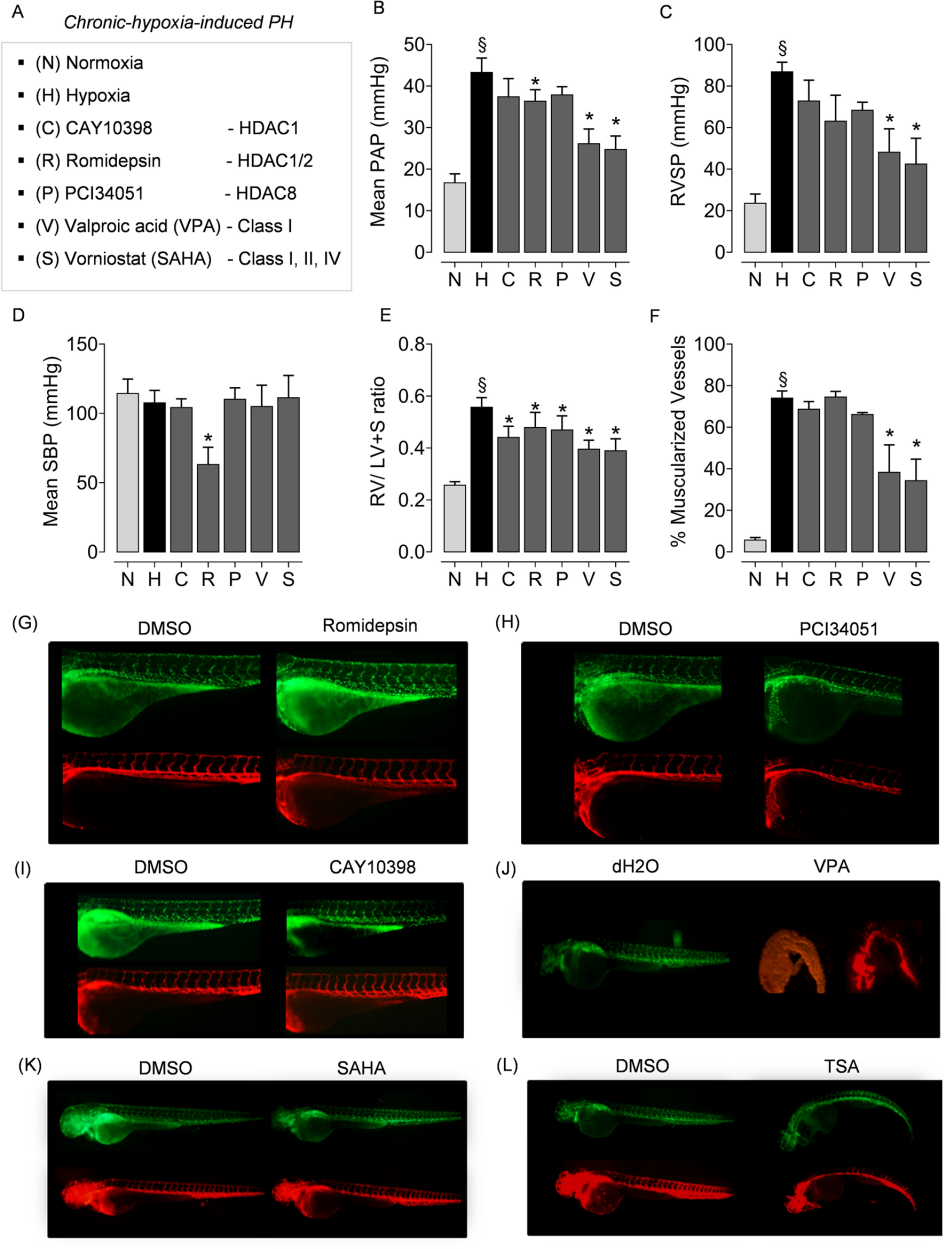
Evaluation of isoform-selective HDAC inhibitors in chronic hypoxia-induced PH and RV hypertrophy in vivo. (**A**) Rats were divided into seven groups and exposed to, (1) normoxia (N), (2) hypoxia (H) for 4 weeks, and hypoxia plus (3) CAY10398, (4) PCI34051 (P), (5) VPA (V), (6) SAHA (S) and (7) Romidepsin (R). Compounds were administered during the last 2 weeks of 4 week hypoxia exposure (n = 6 rats were assigned per group treated with isoform-selective inhibitors). At the end of experiment, haemodynamic parameters were measured and analysed in the animals that completed the treatment regimen. The parameters analysed were (**B**) mean pulmonary artery pressure (mPAP), (**C**) right ventricular systolic pressure (RVSP), (**D**) systolic blood pressure, (SBP), (**E**) right ventricular hypertrophy (RV/LV + septum), and (**F**) percentage of muscularised vessels. Data are represented as bar charts (n ≥ 3; *p < 0.05 versus hypoxia, ^§^p < 0.05 for hypoxia versus normoxia, one-way ANOVA followed by Tukey's multiple comparisons test). To study the effect of HDAC inhibitors on angiogenesis in vivo, the zebrafish embryos were treated with different concentrations of isoform-selective HDAC inhibitors (**G**) Romidepsin (1 µM), (**H**) PCI34051 (100 µM), (**I**) CAY10398 (100 µM), (**J**) class-selective inhibitor VPA (10 mM), and pan-HDAC inhibitors such as (K) SAHA (100 µM), (**L**) TSA (1 µM) or their solvents (DMSO or dd.H2O) 19 hours post fertilization (hpf). HDAC inhibitors were administered to zebrafish embryos at 19 hpf. More than 25 embryos per group were screened for each inhibitor. The zebrafish line employed in this study was generated from Tg(fli1a:nEGFP)y7 (engineered to exhibits green fluorescence), and Tg(kdrl:HsHRAS-mCherry)s896 (engineered to exhibit red fluorescence) to monitor in vivo vascular network.

To evaluate the therapeutic efficacy of HDAC inhibition in vivo, the ex vivo tested compounds that non-selectively and selectively targets class I HDACs (Fig. 7A) were utilized in the rat model chronic hypoxia-induced PH. The therapeutic effects of HDAC inhibition on the attenuation of PH and associated cardiac as well as vascular remodeling were evaluated by hemodynamic measurements and histomorphometry. In accordance with the previous studies^12^, HDAC inhibition with SAHA and VPA attenuated established PH in rats exposed to chronic hypoxia. Both compounds significantly reduced mean pulmonary arterial pressure (mPAP), RV systolic pressure (RVSP), percentage of vascular muscularization (Fig. 7B, C, F). Like the responses upon pan-HDAC inhibition, only HDAC1/HDAC2 selective inhibitor romidepsin showed a significant reduction of hypoxia-induced increase in mPAP (Fig. 7B). However, romidepsin significantly decreased systolic blood pressure (SBP) and failed to attenuate the hypoxia-induced vascular remodeling (Fig. 7D, F). Commercially available compounds tested for selective HDAC1 inhibition with CAY10398 and HDAC8 inhibition with PCI34051 did not attenuate hypoxia-induced increases in mPAP and RVSP (Fig. 7B, C). Intriguingly, both pan- and all three isoform-selective HDAC inhibitors significantly mitigated RV hypertrophy (Fig. 7E). Overall, in comparison to the therapeutic benefits of pan-HDAC inhibitors in the regression of established chronic hypoxia-induced PH, isoform-selective compounds exerted milder effects in vivo as anticipated.

### Impact of HDAC inhibitors on angiogenesis in vivo

Besides the finding that HDAC inhibitors SAHA and VPA significantly regress the established chronic hypoxia-induced PH in vivo, it is also vital to investigate the impact of these HDAC inhibitors on the remodeling and vascularization of the RV. One of the undesirable consequences previously reported to be caused by non-selective HDAC inhibition is the anti-angiogenic effects of trichostatin A (TSA) on RV after pulmonary artery banding (PAB) in rats^19^. Notably, both HDAC inhibitors SAHA or VPA evaluated in this study did not diminish the number of blood vessels or cause vascular rarefaction in the RV tissues prepared from the rats subjected to chronic hypoxia-induced PH in vivo (Supplementary Fig. 4C). Rather, we found an apparent increase in the number of blood vessels in the RVs from rats exposed to hypoxia alone or in the hypoxic rats treated with HDAC inhibitors. Altogether, HDAC inhibitors SAHA and VPA significantly regressed chronic hypoxia-induced PH (Fig. 7B, C), vascular remodeling (Fig. 7F), and RV hypertrophy (Fig. 7E) without anti-angiogenic effects on the RV (Supplementary Fig. 4C), in the experimental model of chronic hypoxia-induced PH in vivo.

Furthermore, to investigate the effects of HDAC inhibition on angiogenesis and in vivo toxicity, we used a vascular cell-specific transgenic zebrafish reporter line as a model system. Zebrafish embryos were treated with different concentrations of pan-HDAC and isoform-selective HDAC inhibitors (Fig. 7A). Notably, SAHA and isoform-selective inhibitors (CAY10398, Romidepsin, PCI34051) did not cause morphological disturbances in angiogenesis and was well tolerated at tenfold higher than concentrations used (Fig. 5A), and did not cause any apparent morphological disturbances during intersegmental vascularization (ISV; Fig. 7G–I,K). Whereas, treatment with TSA exhibited disturbances in the development of ISV compared to solvent-treated controls (Fig. 7L). Surprisingly, the structurally unrelated HDAC inhibitor VPA exerted disruptive effects on the total development of zebrafish (Fig. 7J). This is in accordance with earlier reports of global retardation of development and cardiac teratogenic effects by VPA in zebrafish and other species^20^. Overall, even in a sensitive model of developmental angiogenesis, this experimental study revealed that pan-HDAC inhibitor SAHA and all three isoform-selective inhibitors did not exert any adverse anti-angiogenic effects.

## Discussion

Despite the increased understanding of the pathomechanisms involved in the pathogenesis of PAH and the recent therapeutic advances, PAH remains an incurable disease. Growing evidence emphasizes that the pathogenesis of PAH is linked with abnormalities in epigenetic mechanisms such as DNA methylation, dysregulation of enzyme families, and reader proteins associated with histone acetylation^3^. Most importantly, a recent study demonstrated significant alterations in the global histone acetylation levels in PA endothelial cells (PAEC) isolated from the patients with PAH^21^, which has once again highlighted the dominant role for the regulators of histone acetylation in PAH pathogenesis. Although major attention was given towards therapeutic modulation of HDACs in the context of PH by employing pan-HDAC inhibitors^3^, to circumvent the adverse off-target effects reported on pan-HDAC inhibition, precise identification of dysregulated isoforms in the PAH setting is indispensable to design isoform-selective targeting strategies.

Here, we comprehensively report site specific regulation of class I HDAC isoforms, specifically upregulation of HDAC8 in IPAH-lungs, HDAC1 and HDAC8 in remodeled PAs and HDAC1, HDAC2 and HDAC8 upregulation in PAAFs of IPAH patients. Notably, among four class I HDAC isoforms, HDAC8 transcript and protein levels were consistently increased in various tissues and cells (human lungs, PAs and PAAFs) from patients with PAH. The protein levels of HDAC2 in IPAH-lungs was consistently downregulated, which is in strong agreement with the previous observations reported in IPAH-lungs (Zhao et al.^12^). Interestingly, HDAC8 was significantly differentially expressed and clearly showed a dynamic expression pattern upon exposure to hypoxia ex vivo. Contrary to the ubiquitous upregulation of HDAC1 in remodeled PAs, predominant expression of HDAC2 and HDAC8 is largely colocalized with α-SMA expressing cells in IPAH-PAs with severe medial wall remodeling. This suggests a close association of HDAC2 and HDAC8 with medial hypertrophy and remodeling of the pulmonary vasculature, and warrants further investigation into their biological roles in PH. The expression pattern of HDAC8 corroborates with a previous report that HDAC8 is expressed in vascular SMCs and directly interacts with α-SMA^22^.

Furthermore, among class I HDACs, only HDAC2 transcripts were upregulated in RV tissues from IPAH patients. Previous reports implicate HDAC2 as a positive regulator of hypertrophic response during embryonic development and in the adult heart^23^. Importantly, enzymatic activation of HDAC2 preceded the hypertrophic events, implying that activation of HDAC2 is the cause of cardiac hypertrophy and not a consequence^24^. As disease severity and survival are strongly associated with RV function in patients with PAH, studies employing cardiac-specific knockout of HDAC2 may provide novel insights on HDAC2 mediated RV dysfunction.

Notably, similar to our observations in IPAH, pathophysiological stimuli such as hypoxia indeed plays a major role in the regulation of the critical balance between deacetylase and acetyltransferase activities. Aberrant expression of HDAC and HAT enzymes under hypoxia would subsequently cause a massive imbalance in histone acetylation and deacetylation of lysine residues in core histone tails, which may consequently alter chromatin states and DNA accessibility. In agreement with the time-dependent alterations in the protein levels of deacetylases (HDAC1, HDAC3, HDAC8) (Fig. 6A,B) in response to hypoxia, we also observed time-dependent global changes in the histone modifications (H3K4ac, H3K9/K14ac, and H3K27ac) (Fig. 6C,D) that are typically associated with transcriptional activation. Based on these observations, the hypoxic stress-induced, time-dependent global enrichment or depletion of histone H3 acetylation levels can be directly attributed to the aberrant expression, activity, recruitment, and interplay between acetyltransferases and deacetylases under hypoxia. For instance, acetylation at H3K9/K14 and H3K27 is predominantly deposited by P300/CBP and generally associated with active enhancers and actively transcribed regions^25^. Notably, such time-dependent dynamic shift in global histone acetylation levels induced by hypoxia may ultimately favor cellular hypoxic responses, specifically HIF-dependent transcription of hypoxia-responsive genes^26^. This is of importance, as previous evidence support the notion that PAAFs have a greater propensity to proliferate under hypoxic conditions than resident PASMCs^27,28^, consequently leading to vessel wall thickening and luminal occlusion. Evidently, chronic hypoxia plays a key pathogenic role in PH associated with different chronic lung diseases and hypoxia-induced PH is regarded as a distinct diagnostic entity by the World Health Organization^29^. Our observations emphasize that hypoxic modulation of global histone acetylation significantly imparts alterations in the epigenetic landscape that ultimately favors HIF-dependent transcription of hypoxia-responsive genes and hypoxia-driven phenotypic alterations in vascular cells.

In accordance with expression analysis in IPAH-PAAFs, overexpression as well as knockdown of individual class I HDAC isoforms augmented cellular phenotypes of adventitial fibroblasts. These findings suggest that class I HDAC isoforms regulate the hyperproliferative potential of IPAH-PAAFs and IPAH-PASMCs (Supplementary Fig. 5B,C), suggesting their possible association with medial and adventitial remodeling. Particularly, the transcriptomic analysis revealed a distinct subset of genes and pathways were differentially regulated by HDAC1, HDAC2, and HDAC8 (Fig. 4E). Given the fact that inflammation plays a key role in the pathogenesis of PAH^30^, the signaling pathways regulated by HDAC1 [regulation of viral transcription (GO:0046782); interleukin-6-mediated signaling pathway (GO:0070102)], HDAC2 [positive regulation of NF-κB transcription factor activity (GO:0051092); positive regulation of inflammatory response (GO:0050729); regulation of interleukin-23 production (GO:0032667)], and HDAC8 [positive regulation of immune response (GO:0050778); cellular response to interleukin-1 (GO:0071347)] (Supplementary Table 4), reaffirms the involvement of HDACs in the regulation of inflammatory mediators and immune mechanisms in PAH.

Importantly, identification of genome-wide transcriptional targets regulated by HDAC1, HDAC2, and HDAC8 isoforms in PAH revealed that class I HDAC isoforms regulate a distinct subset of genes. However, the majority of the targets were downregulated upon HDAC knockdown, which is in agreement with the previous reports^31^. Previous studies also reported distinct and redundant regulatory functions of HDAC1 and HDAC2 for proliferation, cell survival, and differentiation, but are also known to act as negative and positive regulators of gene expression^32^. For instance, downregulation of HDAC2 by RNA-interference in IPAH PAAFs significantly downregulated YAP1 transcription, whereas further upregulated LEF1 transcription.

Interestingly, we identified that individual class I HDAC isoforms regulate molecules related to Wnt/β-catenin (LEF1), Hippo (YAP1), flow-responsive (KLF2) and insulin signaling (GLUT4) pathways, which plays an important role in driving PAH pathogenesis. For example, flow-induced transcription factor KLF2, a major regulator of vascular homeostasis is downregulated in IPAH^17^, while a missense mutation was identified in heritable PAH^33^, suggesting a key role for KLF2 signaling in PAH pathogenesis. In agreement with these findings, we also observed decreased KLF2 transcription in hyperproliferative IPAH-PAAFs compared to donors. Importantly, downregulation of HDAC8 by RNA-interference or using pan-HDAC or isoform-selective HDAC inhibition (Fig. 4F, 5G), significantly increased KLF2 transcription in IPAH-PAAFs. Similarly, we also observed that RNA-interference mediated downregulation of HDAC8 increased the transcription of transcription factor retinoic acid receptor alpha (RARα) (Supplementary Fig. 2D).

Regulation of glucose transporter type 4 (SLC2A4 or GLUT4) expression and translocation is critical for glucose homeostasis and metabolic control, and overexpression of GLUT4 reverses insulin resistance^34^. HDAC2 inhibits insulin response of the phosphoinositide 3‐kinase pathway via deacetylation of insulin receptor substrate 1 (IRS1), which inhibits GLUT4 translocation^35^. The depletion of HDAC2 via RNA-interference or HDAC inhibitors is known to partially restore insulin signaling and normal responsiveness to insulin by acetylation of insulin receptor substrate 1 (IRS1)^36^. In accordance, we observed strikingly upregulated GLUT4 transcription upon treatment of IPAH-PAAFs with either pan-HDAC or HDAC1/2 selective inhibitors, which hints the prospect of modulating GLUT4 levels with HDAC inhibitors to counteract the hyperglycemia and insulin resistance mechanisms observed in patients with PAH^37^.

Modulation of HDAC activity remains a prospective therapeutic strategy in several human diseases. Until now, at least four HDAC inhibitors have been approved by the United States Food and Drug Administration (Vorinostat in 2006, romidepsin in 2009, belinostat in 2014, and panobinostat in 2015) for targeted treatment of different cancers^38^. In the case of PAH, we observed promising therapeutic effects of both pan-HDAC and isoform-selective HDAC inhibitors on the reversal of hyperproliferative and apoptosis-resistant phenotype exhibited by PAAFs isolated ex vivo. Additionally, this was further supported by gene-specific modulation using RNA-interference of HDAC isoforms, which confirmed that HDAC isoforms are indeed associated with the disease phenotypes exhibited by IPAH-PAAFs, at variable degrees. This confirms that regulators of histone acetylation play a pivotal role in the maintenance of the phenotype abnormalities exhibited by vascular cells in PAH, which can be reversed upon therapeutic modulation. As expected, HDAC inhibitors SAHA and VPA exerted stronger therapeutic effects and significantly attenuated established PH compared to the isoform-selective HDAC inhibitors, with the exception of RV hypertrophy. These findings suggest involvement of other classes of HDACs in PAH pathogenesis. In line, previous studies have demonstrated the potential role of class IIA HDACs in PAH pathogenesis^12,39,40^. Therefore, future studies are warranted to integrate these studies to provide a greater understanding of the contribution of different classes of HDACs in PAH. On the other hand, despite the significant impact of isoform-selective HDAC inhibitors on the reversal of hyperproliferative and apoptosis-resistant cellular phenotypes on IPAH-PAAFs, the milder therapeutic effects observed in vivo with isoform-selective compounds are acceptable considering the harmful effects imparted by certain non-selective inhibitors like TSA.

Although multiple studies have independently reported the beneficial effects of SAHA and VPA in different preclinical models associated with RV hypertrophy and dysfunction^41^, the promising therapeutic benefits observed with HDAC inhibition was kept under scrutiny due to the anti-angiogenic effects reported with TSA treatments in RV from PAB model^19^. With regards to the compounds used in this study, the previous study reported that VPA neither increased fibrosis nor reduced capillary density^19^, while SAHA was never evaluated for its impact on the vasculature of the RV.

With regards to the general impact of HDAC inhibitors on angiogenesis, SAHA and isoform-selective inhibitors did not cause morphological disturbances even in a sensitive model of developmental angiogenesis in vivo. However, TSA treatment exerted disturbances during the embryonic development of inter-segmental vessels, which perhaps correlate with the anti-angiogenic effects previously observed with TSA^19^. Regarding the developmental defects observed with VPA, our observations are in accordance with previous findings of global retardation of development and cardiac teratogenic effects by VPA in zebrafish and other species^20^. However, in the in vivo experimental setting of chronic hypoxia-induced PH, both pan-HDAC inhibitors SAHA or VPA did not exert anti-angiogenic effects or vascular rarefaction. Under chronic hypoxia, the number of blood vessels was maintained in the RVs, with or without HDAC inhibition. On the contrary, there was an upward trend in the number of blood vessels in the RVs of SAHA and VPA treated rats, which is in accordance with the pro-angiogenic effects of HDAC inhibitors reported by few studies^42,43^. For instance, previous reports demonstrate that both SAHA and VPA treatment enhances endothelial cell sprouting angiogenesis in vitro^42^, while chronic VPA treatment enhances angiogenesis and promotes functional recovery after brain ischemia in vivo via transcription factor HIF1A and its downstream proangiogenic factors^43^. The contrasting reports on pro-angiogenic^43^ or anti-angiogenic effects^19^ of HDAC inhibitors reiterate that the therapeutic outcome and the off-target effects of small-molecule compounds seem to be highly context-dependent, specifically the treatment duration, concentration, and the disease models employed must be prudently evaluated and established in further pre-clinical studies.

Importantly, our in vitro and ex vivo studies suggest that isoform-selective targeting of HDAC8 may perhaps be a potential therapeutic target due to the selective upregulation of HDAC8 in the remodeled pulmonary vasculature of IPAH patients, IPAH-PAAFs and upon hypoxic exposure, while reduction of HDAC8 levels attenuated hyperproliferative phenotype of IPAH-PAAFs ex vivo. However, novel small-molecule inhibitors targeting HDAC8 should be identified and evaluated in multiple rodent models of PH for optimal dosage, route, and time of administration for achieving maximal therapeutic benefit.

Besides HDACs, the importance of histone acetylation pathway in PH pathogenesis is exemplified by the upregulation of acetylation reader protein bromodomain containing 4 (BRD4) in PAH as well as the therapeutic reversal of PH in vivo by pharmacological inhibition of BRD4^43,44^. Finally, the identification of dysregulated HDAC isoforms in PAH, and their transcriptional targets (Supplementary Fig. 2E) have paved a way to specifically target the acetylation pathway using isoform-selective HDAC inhibitors ex vivo and in vivo*.* Thus, we propose that isoform-selective HDAC inhibition is still a worthwhile approach to conduct further studies alone or in combination with contemporary PAH standard of care, in the end, to abrogate disease phenotypes associated with pathological vascular remodeling process in PH.

## Methods

### Human cardiopulmonary tissues

Human lung homogenates, pulmonary artery homogenates, pulmonary artery adventitial fibroblasts (PAAF) for gene and protein expression profiling and functional assays were obtained from explanted lung tissues from subjects with IPAH, which were resected immediately before lung transplantation. Similarly, donor lung samples were taken from downsized lung tissues.

### Human pulmonary vascular cells

Human pulmonary artery adventitial fibroblasts (PAAF) were isolated from human adult donor or IPAH pulmonary arteries. This protocol is approved by the Justus-Liebig-University Giessen Ethics Committee. Segments of pulmonary artery were cut to expose the luminal surface. The intima was removed by gentle scraping with a scalpel blade, and the media was peeled away from the underlying adventitial layer. The adventitial explants were cut into ~ 1 mm^2^ to 2 mm^2^ sections, transferred to T75 flasks and allowed to adhere. Once the cells migrated from tissues and adhere to the flasks, the spots of adventitial cells were further passaged (P) into confluent monolayers and stored in liquid nitrogen freezers. Their phenotype was characterized by gene, protein expression, and immunostaining with fibroblast cell-specific markers. PAAFs were maintained under normoxic conditions (21% O_2_) at 37 °C in a humidified incubator with 5% CO_2_. Importantly, poly-L-Lysine (PLL, ScienCell Research) was used as a coating agent to promote PAAF adhesion in culture. Appropriate amount of water and PLL solution (recommended coating concentration is 2 μg/cm^2^) was added to each cell culture flask or 10 cm dishes and incubated for a minimum of 2 hour (h) at 37 °C. Post-incubation, the PLL solution was removed and rinsed with sterile water before addition of media and cells to the flask.

All primary cells were maintained and expanded in appropriate growth medium to selectively support growth of specific vascular cells. For experiments, human primary vascular cells derived from donors (pulmonary artery smooth muscle cells (PASMC), and pulmonary artery adventitial fibroblasts (PAAF)) were purchased and cultured according to the supplier’s instructions. Once the culture reaches 70% confluence, media was changed on alternative days until the culture was approximately 90% confluent. Vascular cells between fifth to seventh passages were used in transient transfection and hypoxia experiments.

### Laser-assisted microdissection of pulmonary vessels

Laser-assisted microdissection was performed on human lungs from explanted lung tissues from subjects with IPAH (n = 8) or control donors (n = 8) for profiling gene expression. Microdissection steps were performed as described previously^45^. Human lung cryosections (10 μm) were mounted on membrane-coated slides, briefly (45 seconds (s)) stained with hemalaun followed by hematoxylin/eosin, dehydrated in graded ethanol (70%, 96%, 100%), xylene and air dried. Microdissection of intrapulmonary arteries with 50 µm to 100 µm diameter was performed using the laser microdissection device Leica LMD7000 (Leica, Wetzlar, Germany), the microdissected material was collected into RNA lysis buffer, and stored in liquid nitrogen until analysis.

### RNA extraction and cDNA synthesis

Total RNA was extracted from the lung tissues, pulmonary arteries, MDVs and vascular cells using the RNeasy Mini Kit (Qiagen, Germany). Cultured vascular cells or cell pellets were lysed directly by addition of RLT buffer (10 μL of β-Mercaptoethanol/mL of RLT). Whereas, human pulmonary tissues (30 mg) were homogenized in RLT lysis buffer using Precellys ceramic beads (diameter of 1.4 mm; Peqlab Biotechnology, Erlangen, Germany) with Bertin Precellys 24 (Bertin Technologies) homogenizer at 6,000 RPM for 20 s repeated 3 times or until visible tissue was dissolved. Purified RNA from right ventricle was provided by Dr. Hunter C. Champion (University of Pittsburgh, Pennsylvania, USA). Complementary DNA (cDNA) was synthesized from 1 µg of the total RNA using ImProm-II Reverse Transcription System (Promega, Belgium) according to manufacturer’s protocol. cDNA yield was determined by measuring A260 on the Thermo Scientific™ NanoDrop™ 2000 spectrophotometer.

### Real-time quantitative PCR

Real-time quantitative PCR (qPCR or RT-PCR) is widely used for indirect quantification of mRNA levels for the genes of interest. qPCR was performed on a C1000 Thermal cycler (BioRad, USA) using cDNA, gene-specific primers, and iTaq Universal SYBR Green Supermix (Bio-rad, USA). Intron-spanning human-specific primers (Supplementary Table 1) were designed and purchased from Metabion International AG (Germany). Gene expression was analyzed with ΔC_t_ method. The cycle threshold (C_t_) values of the target genes were normalized to the values of the housekeeping gene (endogenous control)_._

### Whole cell protein isolation and estimation

Whole cell lysates were prepared by washing (1X PBS) and suspending the treated cells with cold RIPA lysis buffer (25 mM Tris HCl pH 7.6, 150 mM NaCl, 1% NP-40, 1% sodium deoxycholate, 0.1% SDS) supplemented with 1X Thermo Scientific Halt Protease and Phosphatase Inhibitor Cocktail. Human pulmonary tissues (30–100 mg) were homogenized in RIPA lysis buffer (Thermo Scientific Halt Protease and Phosphatase Inhibitor Cocktail) using Precellys ceramic beads (diameter of 1.4 mm; Peqlab Biotechnology, Erlangen, Germany) with Bertin Precellys 24 (Bertin Technologies) homogenizer at 6000 RPM for 30 s repeated 3 times or until visible tissue was dissolved. The RIPA lysates were centrifuged for 30 minutes (min) at 4 °C (13,000 ×*g*). Supernatants were transferred to 1.5 mL tubes, quantified and stored at − 80 °C. Protein quantification was carried out using Bio-Rad DC Protein Assay kit and Microplate Assay Protocol mentioned in the DC Protein Assay Instruction Manual. Nuclear and cytosolic extracts were prepared from the cells using EpiQuik Nuclear Extraction Kit I (Epigentek, USA).

### Immunoblotting

Whole cell, cytoplasmic and nuclear extracts were resolved in 7.5% or 10% or 15% sodium dodecyl sulfate (SDS)-polyacrylamide gel electrophoresis (depending on the size of proteins). The membranes were blotted with specific primary antibodies (Supplementary Table 2) against HDACs, histone modifications and loading controls that were prepared in blocking buffer and incubated overnight at 4 °C. The membrane was then washed with 1X TBST and further probed with appropriate secondary antibodies conjugated with horse radish peroxidase (HRP). The blot was washed extensively with 1X TBST and developed using an enhanced chemiluminescent (ECL) substrate (SuperSignal West Femto Maximum Sensitivity Substrate, Thermo Scientific, USA). ImageQuant LAS 4000 (GE Healthcare) has high sensitivity and is designed to capture the chemiluminescent signals from the immunoblots. ImageQuant LAS 4000 shows a linear response for chemiluminescent detection with low noise and a wide dynamic range. Western blots were quantified using ImageJ (NIH, Bethesda, MO, USA). Relative protein expression levels were calculated after normalization versus internal controls (housekeeping genes such as GAPDH or β-actin) using intensity values (arbitrary units).

### Immunofluorescence

Paraffin-embedded lung tissue sections (3 μm thick) were deparaffinized in xylene and rehydrated in a graded ethanol series and washed with PBS (pH 7.2). Antigen retrieval was performed by pressure cooking in one of these buffers: Tris buffer (pH 10.0), EDTA buffer (pH 8.0), citrate buffer (pH 6.0) for 15 min. Double immunofluorescence staining was performed with primary antibodies to HDAC1, HDAC2 and HDAC8 (Supplementary Table 2). The same sections were co-stained with vascular cell-specific markers such as α-actin (1:400, C6198, Sigma-Aldrich) for smooth muscle cells, vWF (1:400, IS527, Dako, Germany) for endothelial cells and collagen type I (1:100, T40777R, Meridian Life sciences) for adventitial fibroblasts. After overnight incubation, slides were washed and incubated with the respective secondary antibodies, Alexa Fluor 488-conjugated goat anti-rabbit or anti-mouse or anti-goat IgG (1:1,000, Molecular Probes, Thermo Fisher Scientific Inc.) for 1 hr. Negative control staining with isotype control IgG was also included. All sections were counterstained with DAPI (1:1,000) and mounted with fluorescent mounting medium (Dako). The immunostainings were examined with Leica DM6000B (Wetzlar, Germany).

### Immunocytochemistry

Human donor and IPAH PAAFs were grown to subconfluence in chamber slides, fixed with either acetone-methanol (1:1) or 4% PFA and permeabilized with 0.5% Triton X-100 (Sigma-Aldrich). After blocking (5% BSA in PBS, 1 h), cells were incubated with primary antibodies (HDAC1, HDAC2, HDAC8), followed by Alexa Fluor 488-conjugated secondary antibody (1:1,000) for 1 hr. Negative controls were run in parallel with isotype control IgG (rabbit or mouse or goat) and no primary antibody. The cells were also co-stained for collagen type I (T40777R, Meridian Life sciences) or α-vimentin-Cy3 (1:200, C9080, Sigma). After incubation, slides were counterstained with DAPI (for nuclear staining) and mounted with fluorescent mounting medium (Dako). Images were taken at 400 × magnification with Leica DM6000B (Wetzlar, Germany).

### Histone extraction

Human PAAFs were harvested, pelleted and washed twice with ice-cold PBS. Histones were isolated from the cell pellets with EpiQuik Total Histone Extraction Kit (Epigentek, NY) according to the manufacturer’s instructions. Briefly, samples were homogenized with 1 × pre-lysis buffer (10 min, on ice), and centrifuged at 10,000 RPM for 1 min at 4 °C. The supernatant was removed and re-suspended in 3 volumes (approximately 200 µL/10^7^ cells) of Lysis Buffer and incubated on ice for 30 min. The samples were centrifuged at 12,000 RPM for 5 min at 4 °C. The supernatant fraction was transferred into a new vial and supplemented with 0.3 volumes of the Balance-DTT Buffer. The protein concentration was measured with Bio-Rad DC Protein Assay kit and stored at -20˚C until further use.

### HDAC activity assay

Total HDAC activity in donor and IPAH PAAFs was quantified with Epigenase HDAC Activity/Inhibition Assay Kit, according to the manufacturer’s protocol. The ratio or amount of deacetylated products, which is proportional to the enzyme activity, was colorimetrically measured by reading the absorbance on a microplate reader within 2 to 10 min at 450 nm with an optional reference wavelength of 655 nm. The activity of the HDAC enzyme is proportional to the OD intensity measured. The HDAC assay standard (deacetylated histones) provided in kit was used for quantification of HDAC enzyme activity. Replicate samples were run to ensure that the signal generated is validated. HDAC activity or inhibition was calculated using the following formula, HDAC Activity (OD/min/mg) = ((Sample OD – Blank OD)/(Protein Amount (µg) × min)) × 1,000.

### Small-molecule inhibitors

The effect of small-molecule inhibitors on vascular cell proliferation, apoptosis, gene and protein expression was assessed with appropriate assays post-treatment. The cells grown to sub-confluence were treated with increasing concentrations of HDAC inhibitors. The concentrations of the compounds were chosen based on previous studies and from the cell viability assays. PAAFs isolated from IPAH patients were subjected to treatment with HDAC inhibitors such as SAHA (pan-HDAC inhibitor, Selleck Chemicals), Trichostatin A (pan-HDAC inhibitor, Sigma-Aldrich), VPA (class I > class IIa HDAC inhibitor^46^, Sigma-Aldrich), isoform-selective CAY10398 (HDAC1 inhibitor, Cayman Chemical Company), PCI34051 (HDAC8 inhibitor, Selleck Chemicals), Romidepsin (HDAC1/HDAC2 inhibitor, Selleck Chemicals). Simultaneously, the cells were also treated with respective concentrations of the solvent (DMSO from Sigma-Aldrich or double distilled water) in parallel.

### Hypoxia exposure

To determine the effect of hypoxia exposure on gene, protein expression and histone modifications, human PAAFs were incubated in 1% O_2_, 5% CO_2_, balance N_2_ (normobaric hypoxia) for different time points 6, 24, 48 h. Normoxic controls were also cultured simultaneously in a humidified incubator at 37 °C in the presence of 5% CO_2_. Post-hypoxia exposure time period, the cells were harvested and analyzed.

### Cell proliferation assay

Monitoring DNA synthesis is an indirect parameter of cell proliferation. BrdU incorporation assay was performed to assess vascular cell proliferation using Cell Proliferation ELISA Kit (Roche Applied Science, Indianapolis, IN), according to manufacturer’s protocol. Briefly, the cells were trypsinized and seeded in poly-l-Lysine coated 96-well plates at a density of 5,000 cells (200 µL/well) in complete growth media for 24 h. The difference in cell proliferation between donor versus IPAH PAAFs was assessed directly by BrdU assay after 24 h. To assess the effect of small-molecule inhibitors on cell proliferation, the inhibitors were added onto the plates already grown to sub-confluence (overnight), incubated for 24 h and then subjected to BrdU incorporation. Similarly, PAAFs or PASMCs were transiently transfected with siRNA or plasmid DNA in 48-well plates and then subjected to BrdU assay, 48 h post-transfection. Post-treatment, the cells were labeled using 10 µM BrdU per well and re-incubated for 2 h at 37 °C in a humidified atmosphere. After removal of culture media, the cells were fixed, and the DNA was denatured in one step by adding FixDenat. Cells were further incubated with the anti-BrdU-POD antibody for 90 min at room temperature. The cells were washed and the immune complexes were detected by addition of substrate solution. The reaction product was quantified by measuring the absorbance using a scanning multi-well spectrophotometer (ELISA reader) at 370 nm with a reference wavelength of 492 nm.

### Apoptosis assay

Apoptotic cell death induced by treatment with inhibitors or siRNA/plasmid transfection was determined by Cell Death Detection ELISA^PLUS^ kit (Roche) using quantitative sandwich-enzyme-immunoassay-principle. Post-treatment, the plates were subjected to the cell death assay according to the manufacturer’s protocol. Briefly, the plates were centrifuged for 10 min, at 200×*g* and the supernatant was removed carefully. The cell pellet was re-suspended in 100–200 µL Lysis buffer, incubated for 30 min at 15–25 °C and centrifuged at 200×*g* for 10 min. 20 µL of supernatant (cytoplasmic fraction) was carefully transferred to streptavidin coated plate to which 80 µL of the immunoreagent was added further and incubated on a MP shaker under gently shaking (300 RPM) for 2 h at 15–25 °C. The plate was washed three times with 250–300 µL incubation buffer, before incubation with 100 µL ABTS solution for color development. The plates were measured using a microplate reader (Infinite 200 PRO NanoQuant, TECAN at 405 nm against ABTS solution as a blank (reference wavelength approx. 490 nm).

### RNA-interference

Human vascular cells were transfected with isoform-specific siRNAs targeting HDACs or target genes using either Amaxa Basic Nucleofector Kit for Primary Mammalian Fibroblasts or Lipofectamine 3000 Transfection Reagent (Invitrogen), according to the manufacturer’s protocol. As a negative control, commercially available nontargeting siRNA (AllStars Neg. Control siRNA, Qiagen) was used. Additionally, we also performed validation experiments using ON-TARGETplus SMARTpool siRNAs in IPAH-PAAFs. Six hours after transfection, the cells were maintained in complete medium for a resting period of 24 h to evaluate the knockdown efficiency at mRNA and 48 h at protein level. The efficiency of gene knock-down was verified by qPCR or immunoblotting. Post-transfection, we studied the effect of siRNA knockdown on cell proliferation, apoptosis, gene and protein expression with appropriate assays, at specific time points.

### Transient overexpression

Human vascular cells or ECV304 or HEK293 were transiently transfected with specific plasmids overexpressing HDAC1 (Sino Biological Inc.), HDAC2 (received as a gift from Prof. Edward Seto, George Washington Cancer Center), HDAC8 (OriGene Technologies Inc.) or LEF1 (Sino Biological Inc.) using either Amaxa Basic Nucleofecto Kit for Primary Mammalian Fibroblasts or TurboFect Transfection Reagent (Thermo Scientific), according to the manufacturer’s protocol. As a negative control, empty vector plasmids were used. Six hours after transfection, the cells were cultured in complete medium for a resting period of 24 h to evaluate the overexpression at mRNA level and 48 h at protein level. Post-transfection, we studied the effect of HDAC or LEF overexpression on cell proliferation, apoptosis, gene and protein expression with respective assays.

### Microarray-based genome-wide expression analysis

Transcriptome profiling was performed with microarrays (Agilent-039494: SurePrint G3 Human GE v2 8 × 60 K Microarray) following RNA-interference in IPAH-PAAFs, with validated isoform-specific HDAC1, HDAC2 and HDAC8 siRNAs along with scrambled siRNA as a negative control. Heatmaps showing hierarchical clustering of differentially expressed genes in IPAH-PAAFs upon HDAC RNAi versus scrambled treated IPAH-PAAFs (log_2_FC ≤ − 1 or log_2_FC ≥  + 1, FDR < 0.05) were generated. Data are mean ± SD (n = 3, biological replicates). Gene ontology enrichment analysis was performed on the target genes of HDAC isoforms (log_2_FC ≤ − 1 or log_2_FC ≥  + 1, FDR < 0.01) identified from all three siRNA comparisons (IPAH + HDAC1, IPAH + HDAC2, IPAH + HDAC8 siRNA versus IPAH + Scrambled groups) using Enrichr^47^ and results are listed in Supplementary Table 4. The values used for generating heatmaps in Figs. 4F and 5F are provided in Supplementary Table 3.

### Animals and experimental design

Adult male Sprague–Dawley (SD) rats (body weight 200–250 g) (Charles River, UK) were used. Consecutive in vivo experiments were designed as rats were divided into seven groups (n = 6) and exposed to (1) normoxia (N), (2) hypoxia for 4 weeks (H), (3) hypoxia and CAY10398 (10 mg/kg, 3 times per week) (C), (4) hypoxia and PCI34051 (10 mg/kg, 3 times per week) (P), (5) hypoxia and VPA (300 mg/kg/day) (V), (6) hypoxia and SAHA (50 mg/kg/day) (S) and (7) hypoxia and Romidepsin (0.5 mg/kg, twice per day) (R). All the treatments were started after two weeks hypoxia exposure and continued for the remaining 2 weeks of hypoxia exposure. CAY10398 and PCI34051 were dissolved in 1:1:2 DMSO:PEG-300:normal saline while Romidepsin was dissolved in 1:1 DMSO:PEG-300; these three treatments were given by intraperitoneal injection. VPA was dissolved in distilled water and SAHA in 5 molar equivalents 2-hydroxypropyl-β-cyclodextrin (HOP-β-CD, Sigma-Aldrich) as previously described. Animals were weighed every other day and treatment doses were calculated accordingly.

### Hemodynamic measurements, tissue collection and histology

Rats were anaesthetised (Hypnorm 1 mL/kg i.m; Hypnovel 0.8 mL/kg i.p). Right ventricular systolic pressure (RVSP) and pulmonary arterial pressure (PAP) were measured via a pre-curved catheter inserted through the right jugular vein and systemic blood pressure (SBP) recorded in the carotid artery cannulation using a PowerLab Data Acquisition system (ADInstruments Ltd). At the end of experiment, the animals that completed the experiment were then sacrificed; tissues were collected, snap frozen, and stored at − 80 °C for biochemical measurements. Hearts were dissected and weighed, and the ratio of RV to left ventricle plus the septum mass was used as an index of RV hypertrophy [RV/(LV + septum)]. The left lung was fixed with 10% formalin in PBS.

### Zebrafish angiogenesis

To assess the effect of pan- and selective HDAC inhibitors on angiogenesis, zebrafish embryos were treated with different concentrations of commercially available pan-HDAC inhibitors (1 µM TSA, 100 µM SAHA), class I HDAC-selective (10 mM VPA), isoform-selective HDAC inhibitors (100 µM CAY10398, 100 µM PCI34051, 1 µM Romidepsin) or their respective solvents (DMSO, water). HDAC inhibitors were administered to zebrafish embryos at 19 h post fertilization (hpf). Concentration of HDAC inhibitors used for zebra fish experiments were chosen from ex vivo functional experiments with IPAH-PAAFs, in addition concentrations up to 10 times higher concentration was also evaluated. The effect of HDAC inhibitors on vascularization was visually inspected at 6, 12, 24, 48 and 72 h, and fluorescent microscopic images were captured at 24, 48 and 72 h post-treatment for representation (*24 h, **48 h post-treatment). The inhibitor treatment groups were visually compared to the cells treated with vehicle or solvent control (DMSO or dd.H2O). We screened > 25 embryos per group for each inhibitor and each experiment was performed in triplicates. Embryos were maintained in a humidified incubator at 28 °C. The zebrafish lines employed in this study was provided by Dr. Benno Jungblut (Max-Planck-Institute for Heart and Lung Research) and generated from Tg(fli1a:nEGFP)y7 that was engineered to exhibit green fluorescence, and similarly Tg(kdrl:HsHRAS-mCherry)s896 was engineered to exhibit red fluorescence to monitor in vivo vascular network.

### Chromatin immunoprecipitation assay and ChIP qPCR

PAAFs were cross-linked by adding 37% formaldehyde directly to the culture dishes to a final concentration of 1% at room temperature (RT) for 10 min. The cross-linking reaction was quenched with 2.5 M glycine at a final concentration of 125 mM for 5 min at RT and the cells were then washed thrice and scraped into ice-cold 1X PBS. The subsequent steps until crosslink reversal were performed at 4 °C or on ice. The cells were harvested in ice-cold lysis buffer and the nuclei extracts were sonicated using a Bioruptor (Diagenode, Belgium) under optimal shearing conditions to generate fragment size of 200–500 base pairs. After centrifugation, the resultant supernatant fraction of the sonicated samples was diluted and 1% of every sample was stored as input. The samples were precleared with salmon sperm DNA/ protein A agarose-50% slurry (Millipore, USA) for 2 h at 4 °C with rotation (8 RPM). Immunoprecipitation was performed on the precleared chromatin overnight at 4 °C with rotation (8 RPM) with specific anti-acetyl-Histone H4 (Millipore). As negative controls, immunoprecipitation with a non-specific rabbit IgG or no primary antibody was performed. The antibody/histone complexes were recovered by incubating with salmon sperm DNA/protein A agarose-50% slurry for 2 h at 4 °C with rotation (8 RPM) followed by gentle centrifugation at 1,000×*g* for 5 min. Following the washing steps, the immune-complexes were eluted and reverse cross-linked overnight. The reverse cross-linked DNA fraction was then purified using a QIAquick PCR Purification Kit according to the manufacturer's protocol. Quantitative analysis was performed by real-time qPCR using iQ SYBR Green supermix (Bio-rad, USA) and specific primer pairs spanning proximal and distal regions relative to the transcription start site of the target genes. The level of enrichment for the target locus was calculated by analyzing ChIP qPCR data relative to total input DNA fraction (percent input) and the enrichment was compared to pre-immune rabbit IgG (negative control).

### Ethics statement

All human samples used in this study were approved by the ethics committee (Ethik Kommission am Fachbereich Humanmedizin der Justus Liebig Universität Giessen) of the University Hospital Giessen (Germany) in accordance with national law and with Good Clinical Practice/International Conference on Harmonisation guidelines. Written informed consent was obtained from each individual patient or the patient’s next of kin (AZ 31/93, AZ 111/08‐eurIPFreg). All the Zebrafish angiogenesis experiments were carried out in accordance with relevant institutional guidelines and regulations (Max-Planck-Institute for heart and lung research, Germany). All experimental protocols on animals were reviewed and approved by the ethics committee (Animal Welfare and Ethical Review Body (AWERB) of the Imperial College London). All animal experiments were conducted at Imperial College London, UK in accordance with the scientific protocols and methods that was approved under the project licence (PPL) number: 70/7,425, by the UK Home Office regulations, animals in science committee (ASC) under Animals (Scientific Procedures) Act 1986 (London, UK).

### Statistical analysis

Data are calculated as either mean ± SEM with values from biological replicates. Statistical analyses were performed with Student’s t-test for comparisons between two groups with P value < 0.05 considered to be significant. Statistical analysis was not performed for experiments with n = 1 (biological replicate), although they had experimental replicates from same donor (Supplement 3G, 3H, 3I). Information on specific statistical analysis and the number of experimental or technical replicates are indicated in the respective figure legends. All statistical analyses were performed using Prism 6.0 (GraphPad Software, CA, USA).

## Supplementary information

Supplementary Information 1.

Supplementary Table 1.

Supplementary Table 2.

Supplementary Information 2.

Supplementary Information 3.

## Abbreviations

PH: Pulmonary hypertension
PAH: Pulmonary arterial hypertension
IPAH: Idiopathic PAH
PAAF: Pulmonary artery adventitial fibroblasts
PASMC: Pulmonary artery smooth muscle cells
RV: Right ventricle
HDAC: Histone deacetylase
HAT: Histone acetyltransferases
SAHA: Suberanilohydroxamic acid
TSA: Trichostatin A
VPA: Valproic acid
ISV: Intersegmental vessels
mPAP: Mean pulmonary arterial pressure
RVSP: RV systolic pressure
SBP: Systolic blood pressure
