# Supplementary material for: Isoform-specific characterization of class I histone deacetylases and their therapeutic modulation in pulmonary hypertension
Source: Sci Rep. 2020 Jul 30;10:12864. doi: 10.1038/s41598-020-69737-x (PMC7393135; doi:10.1038/s41598-020-69737-x)
Supplement: Supplementary file 1 — Supplementary Information 1. [file 41598_2020_69737_MOESM1_ESM.docx]

**Isoform-specific characterization of class I histone deacetylases and their therapeutic modulation in pulmonary hypertension**

Prakash Chelladurai^1^, Swati Dabral^1*^, Shobha Rani Basineni^1*^, Chien-Nien Chen^2*^, Mario Schmoranzer^1^, Nina Bender^1^, Christine Feld^3^, René Reiner Nötzold^3^, Gergana Dobreva^4^, Jochen Wilhelm^5^, Benno Jungblut^1^, Lan Zhao^2^, Uta-Maria Bauer^3^, Werner Seeger^1,5^, Soni Savai Pullamsetti^1,5^

^(1)^ Max-Planck Institute for Heart and Lung Research, Bad Nauheim, Germany; German Center for Lung Research (DZL), Giessen, Germany

^(2)^ Center for Pharmacology and Therapeutics, Experimental Medicine, Hammersmith Hospital, Imperial College London, United Kingdom

^(3)^ Institute of Molecular Biology and Tumor Research, Philipps University Marburg, Germany

^(4)^ Department of Anatomy and Developmental Biology, CBTM, Medical Faculty Mannheim, Heidelberg University, Mannheim, Germany

^(5)^ Department of Internal Medicine, Justus-Liebig-University Giessen, Klinikstrasse 36, 35392, Giessen, Germany

* Equal contribution

Email address of corresponding author: [soni.pullamsetti@mpi-bn.mpg.de](mailto:soni.pullamsetti@mpi-bn.mpg.de)

# Supplementary Data

Supplementary Figure 1: Validation of HDAC antibodies and overexpression constructs

Supplementary Figure 2: RNA-interference of HDAC isoforms in IPAH PAAFs

Supplementary Figure 3: LEF1 is coregulated in PAH, regulated by HDAC2 and promotes PAAF proliferation

Supplementary Figure 4: HDAC inhibition in IPAH PASMCs *ex vivo* and chronic-hypoxia-induced PH *in vivo*

Supplementary Figure 5: RNA-interference of HDAC isoforms in IPAH-PAAFs and PASMCs *ex vivo*.

Supplementary Table 1 List of Primers

Supplementary Table 2: List of antibodies

Supplementary Table 3: Differentially expressed gene lists from microarray analysis

Supplementary Table 4: Gene ontology analysis from microarray data

Supplementary Images (Uncropped Western Blots)

#### Supplementary Figures

####
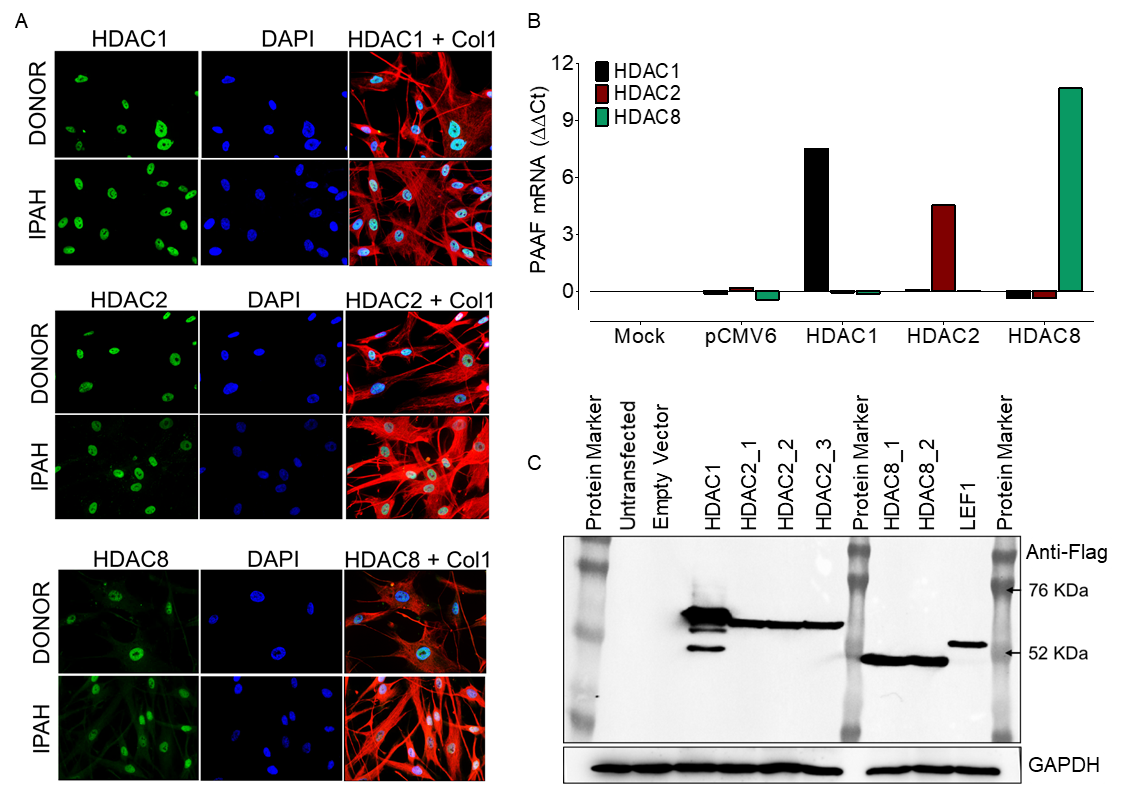


**Supplementary Figure 1: Validation of HDAC antibodies and overexpression constructs.** (A) Subcellular localization of endogenous expression of HDAC1, HDAC2, HDAC8 proteins (Green) in donor-PAAFs and IPAH-PAAF *ex vivo*. The HDAC isoforms were visualized by immunofluorescence microscopy using goat anti-rabbit or anti-mouse Alexa Fluor 488 secondary antibody. Human PAAFs were co-stained with fibroblast cell-specific marker Vimentin conjugated with Cy3 fluorophore (Red), while the nucleus was counter-stained with DAPI. (B) Validation of isoform-specific HDAC overexpression in human donor-PAAFs, which were transiently transfected with HDAC1, HDAC2 and HDAC8 plasmids using Amaxa^TM^ Basic Nucleofector Kit for primary mammalian fibroblasts according to manufacturer’s protocol. Gene expression analysis of overexpressed HDAC isoforms was performed by RT-PCR from the RNA isolated from PAAFs (n=1), 48 hours post-transfection. ∆C_t_ values were calculated using β2M as reference and further normalized (∆∆C_t_) to mock control. (C) Human HEK293 cell line was transiently transfected with HDAC1, HDAC2, HDAC8 and LEF1 plasmids using TurboFect transfection reagent and whole cell proteins were prepared, 48 hours post-transfection and analysed by western blotting using anti-Flag antibody. Empty vector plasmid was used as a negative control.


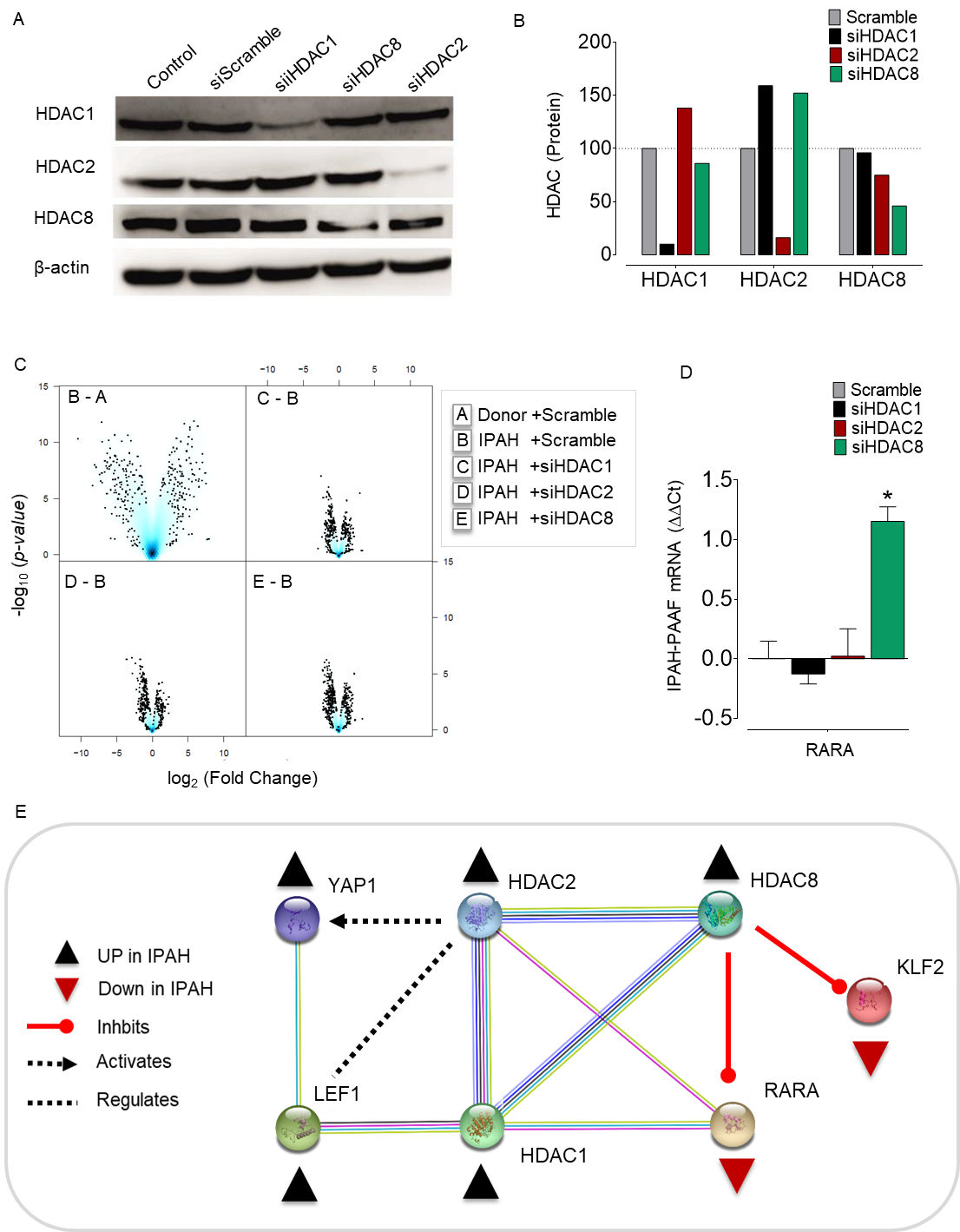


**Supplementary Figure 2: RNA-interference of HDAC isoforms in IPAH-PAAFs.** (A) Validation of isoform-specific knockdown by RNA-interference. Donor-PAAFs were transiently transfected with multiple siRNAs for each HDACs, HDAC1, HDAC2 and HDAC8 using Amaxa Basic Nucleofector Kit for primary mammalian fibroblasts according to manufacturer’s protocol. In addition to untransfected cells (Control), scrambled siRNA was used as negative control. Whole cell proteins were prepared from donor-PAAFs (n=1), 48 hours post-transfection and analyzed by western blotting. (B) The band densities in the immunoblots were further quantified using ImageJ program. β-actin was used as a loading control. (C) Genome-wide identification of transcriptional targets of HDAC isoforms. To identify their respective genome-wide transcriptional targets, transcriptome profiling was performed with microarrays (Agilent-039494: SurePrint G3 Human GE v2 8x60K Microarray) following RNA-interference in IPAH-PAAFs (n=3), with validated isoform-specific HDAC1, HDAC2 and HDAC8 siRNAs along with scrambled siRNA as a negative control. Volcano plot shows the distribution of gene expression with the –log_10_ of p-value (y-axis) is plotted against the log_2_ fold change between 2 groups (x-axis). (D) RNA-interference in IPAH-PAAFs (n=2 biological replicate, 3 experimental replicate each) was performed with validated isoform-specific HDAC1, HDAC2 and HDAC8 siRNAs along with scrambled siRNA as a negative control, and evaluated by RT-PCR. ∆C_t_ values were calculated using β2M as reference and further normalized (∆∆C_t_) to scrambled siRNA-treated IPAH-PAAFs. (E) Functional association network between candidate genes was visualized using STRING database and the regulatory links identified in this study (Figure 4F, 5F) is highlighted on the network representation.


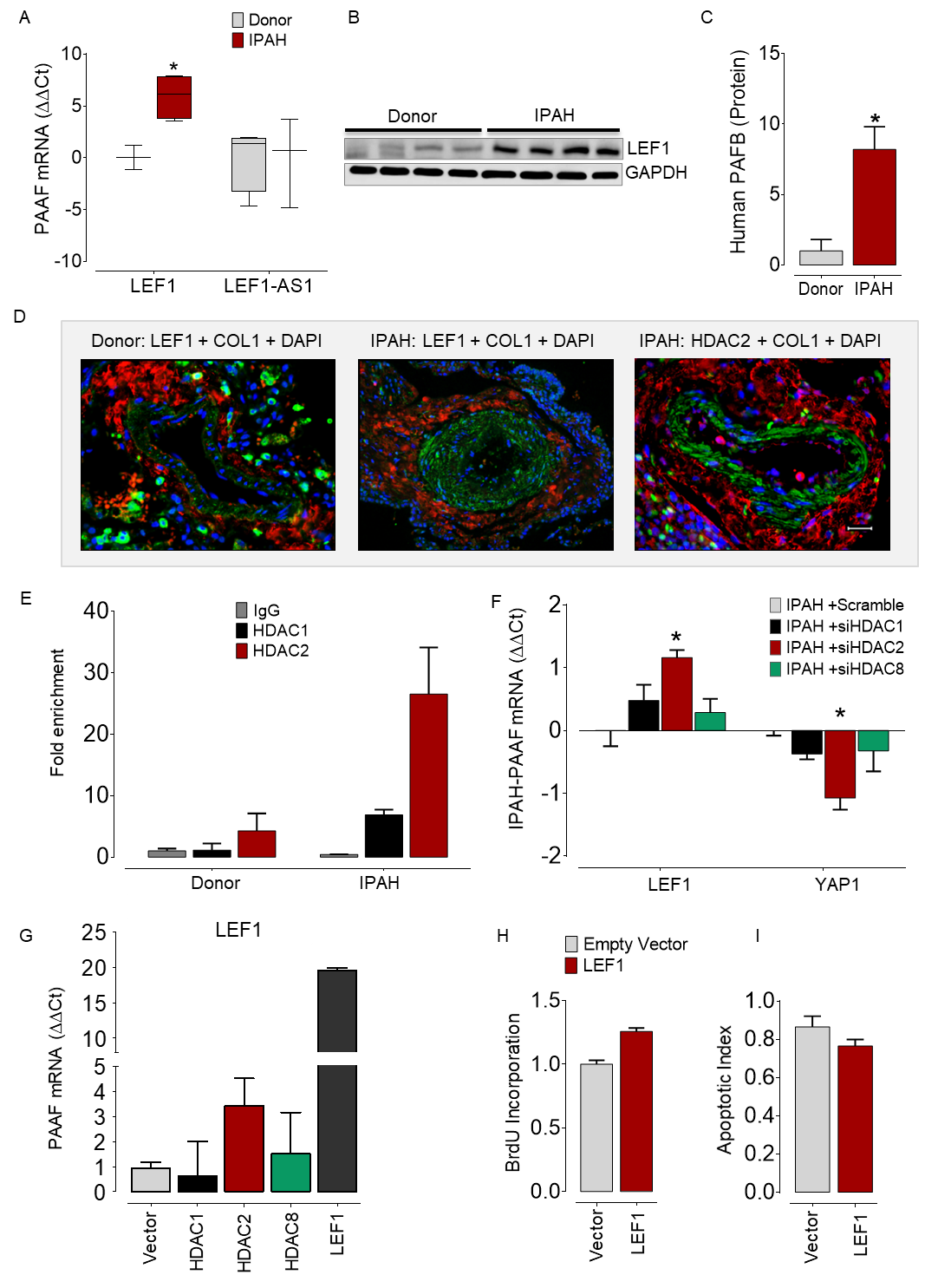


**Supplementary Figure 3: LEF1 is coregulated in PAH, regulated by HDAC2 and promotes PAAF proliferation.** (A) RT-PCR analysis of LEF1 and LEF1-AS1 was performed on the RNA isolated from PAAF of healthy donors (n=4) and IPAH (n=4) patients. ∆C_t_ values were calculated using β2M as reference and further normalized (∆∆C_t_) to donor controls. (B) Western blots were performed on lysates extracted from the human PAAFs that were harvested from healthy donors (n=4) and IPAH (n=4) patients. (C) Blots were quantified by densitometry and are represented as bar charts after normalization to internal loading control. (D) Representative microscopic pictures of human pulmonary arteries immunostained for LEF1 and HDAC2 expression in human donor and IPAH lung sections. LEF1 or HDAC2 (in green fluorescence) expression was localized to collagen I (COL1A1/COL1, Red) associated with adventitial fibroblasts, while the nucleus was counter-stained with DAPI (Blue). Scale bar: 50µm. (E) Chromatin immunoprecipitation (ChIP) was performed with HDAC1 and HDAC2 antibodies on chromatin preparations harvested from donor- and IPAH- PAAFs (n=2). Quantitative real-time PCR was performed with primers specific for LEF1 promoter. ChIP qRT-PCR results were represented as fold enrichment over IgG. (F) RNA-interference in IPAH-PAAFs (n=3) was performed with validated isoform-specific HDAC1, HDAC2 and HDAC8 siRNAs along with scrambled siRNA as a negative control, and evaluated by RT-PCR∆C_t_ values were calculated using β2M as reference and further normalized (∆∆Ct) to scrambled siRNA-treated IPAH-PAAFs, *p<0.05, Student's t-test. (G) Validation of LEF1 overexpression was achieved by transient transfection of donor-PAAFs with validated LEF1 as well as HDAC1, HDAC2, HDAC8 plasmids. RT-PCR analysis was performed on the RNA isolated from donor-PAAFs (n=1, 2 experimental replicates), 24hrs post-transfection. Data was normalized normalized (∆∆C_t_) to empty vector control. (H) Overexpression was achieved by transient transfection of donor-PAAFs (n=1) with validated LEF1 plasmid, and their functional effects on proliferation was assessed by BrdU incorporation and (I) apoptosis, 48 hours post-transfection. Empty vector plasmid was used as a negative control.


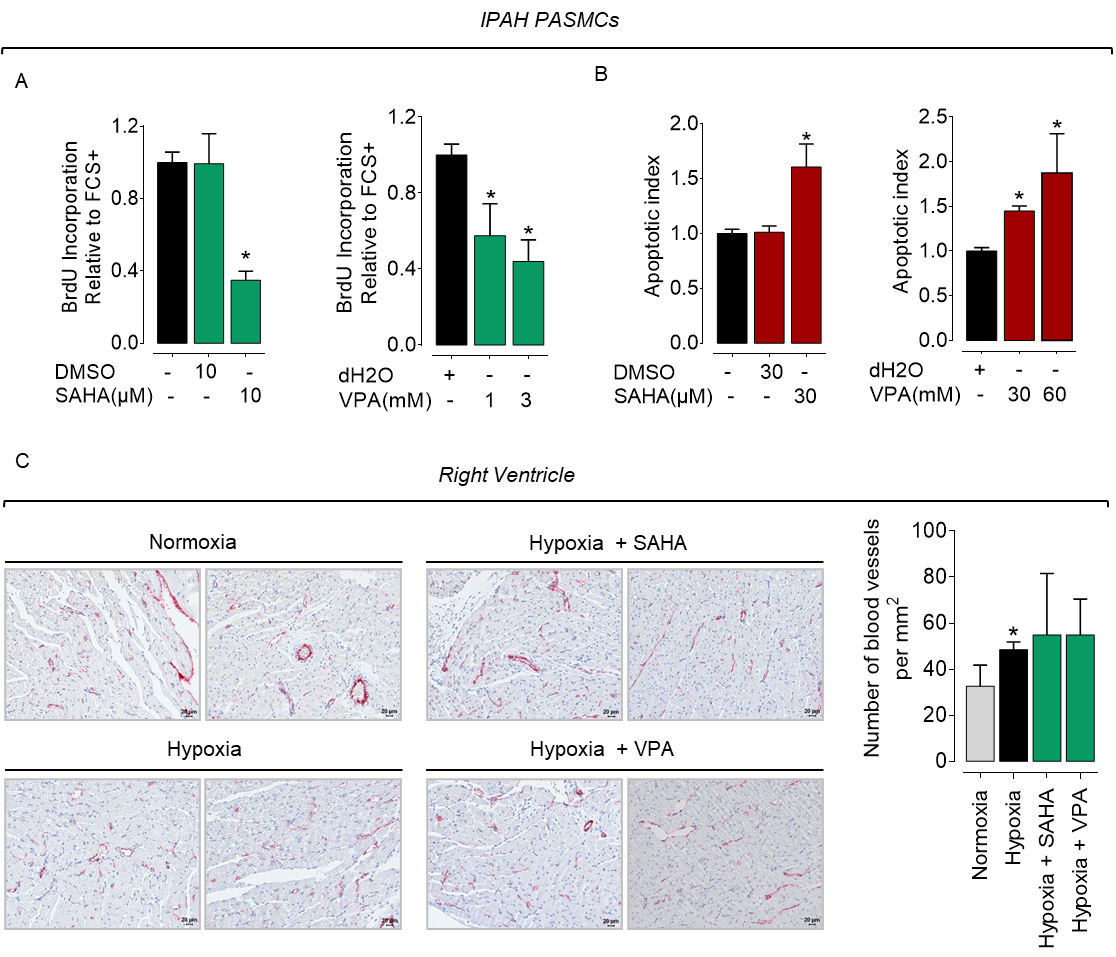


**Supplementary Figure 4: HDAC inhibition in IPAH-PASMCs *ex vivo* and chronic-hypoxia-induced PH *in vivo*.** IPAH-PAAFs were treated with increasing concentrations of commercially available pan-HDAC inhibitor SAHA and VPA. (A) Cell proliferation was assessed by BrdU incorporation and (B) induction of apoptosis was assessed by Cell Death Detection ELISA^PLUS^, 24 hours post-treatment. Absorbance values obtained for HDAC inhibitor and solvent treatments were normalized to the BrdU incorporation of untreated cells. Data are represented as bar plots (n=2; *p<0.05 versus solvent control, Student's t-test). (C) Right ventricle (RV) sections were prepared from the *in vivo* chronic-hypoxia experiment (from Fig. 7E). Representative photomicrographs (Scalebar = 20µM) are shown from the RV sections subjected to immunohistochemical staining for von Willebrand factor (vWF), followed by microscopic quantification of the number of vessels in RV sections per mm^2^.


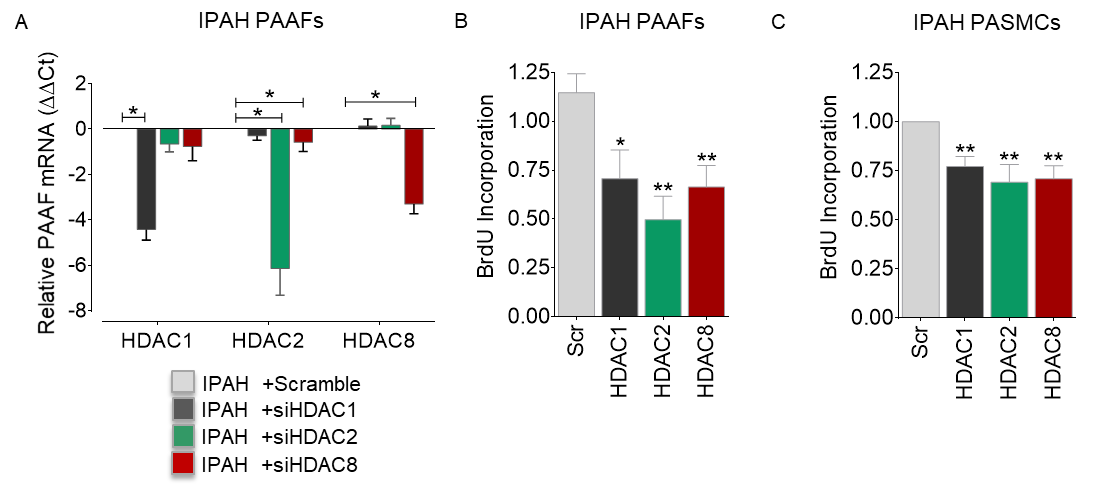


**Supplementary Figure 5: RNA-interference of HDAC isoforms in IPAH-PAAFs and PASMCs *ex vivo*.** RNA-interference was achieved by transient transfection of validated HDAC1, HDAC2 and HDAC8 siRNAs (ON-TARGETplus siRNA) in IPAH-PAAFs cultured *ex vivo*. (A) RT-PCR analysis was performed on the RNA isolated from IPAH-PAAFs (n=3). ∆C_t_ values were calculated using β2M as reference and further normalized (∆∆C_t_) to scrambled siRNA-treated IPAH-PAAFs. Functional effects of RNA-interference on fetal calf serum (FCS) induced proliferation was assessed by BrdU incorporation in (B) IPAH-PAAFs, and (C) IPAH-PASMCs, 48 hours post-transfection. Data was normalized to scrambled-siRNA treated controls (IPAH; n=3; *p<0.05 versus scrambled siRNA, Student's t-test).

## Supplementary Images (Uncropped Western Blots)

**
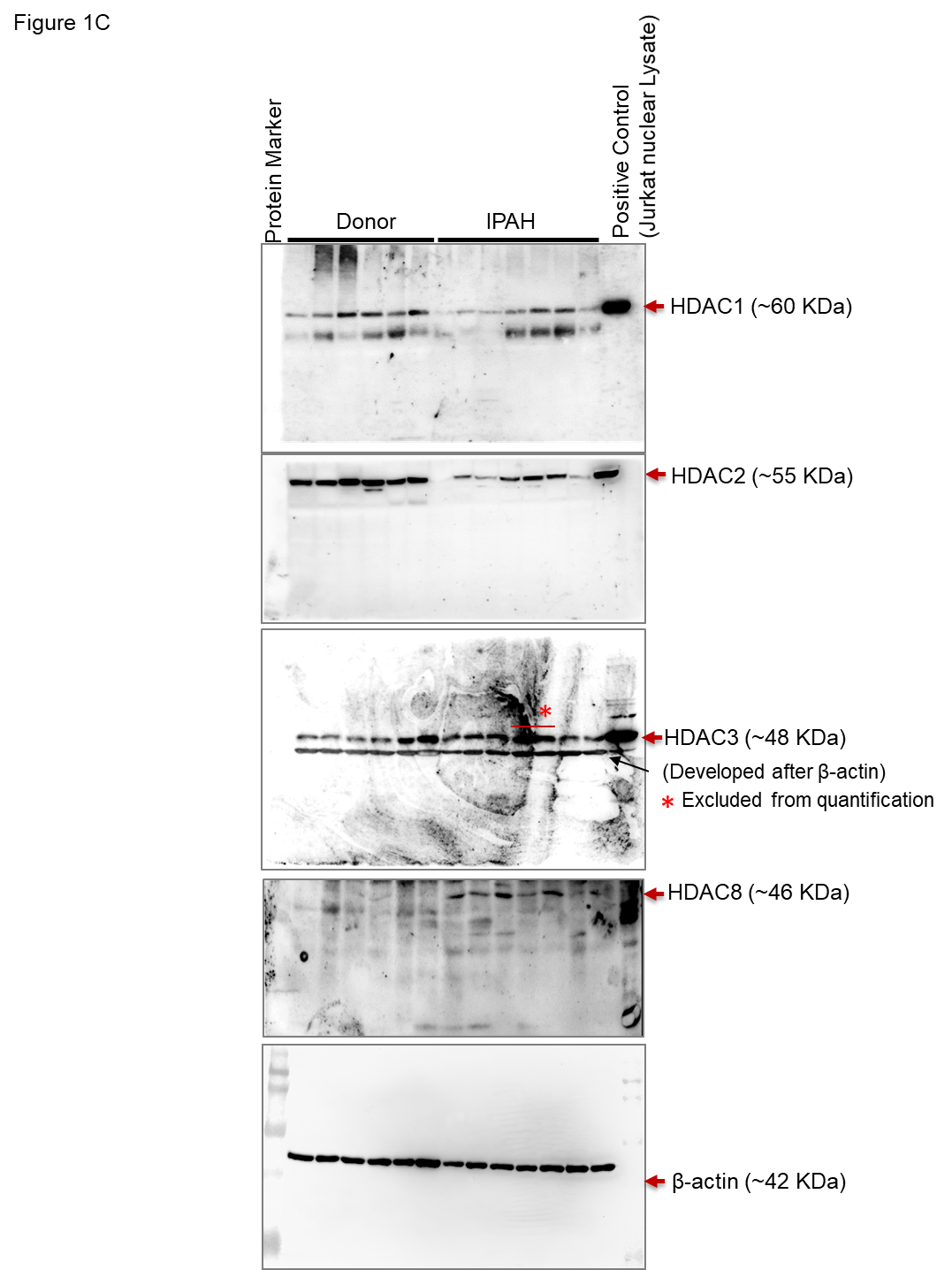
**

**
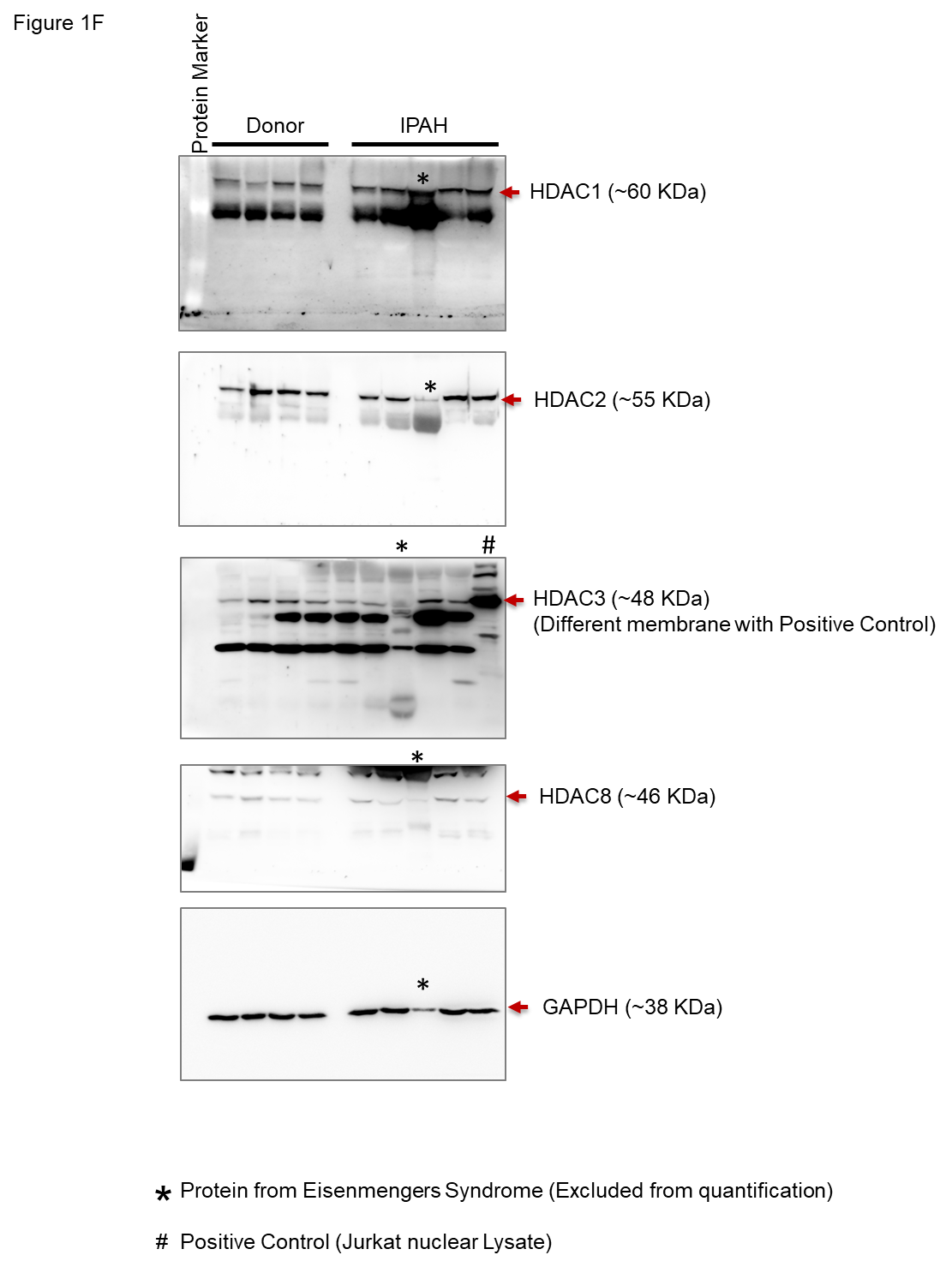
**

**
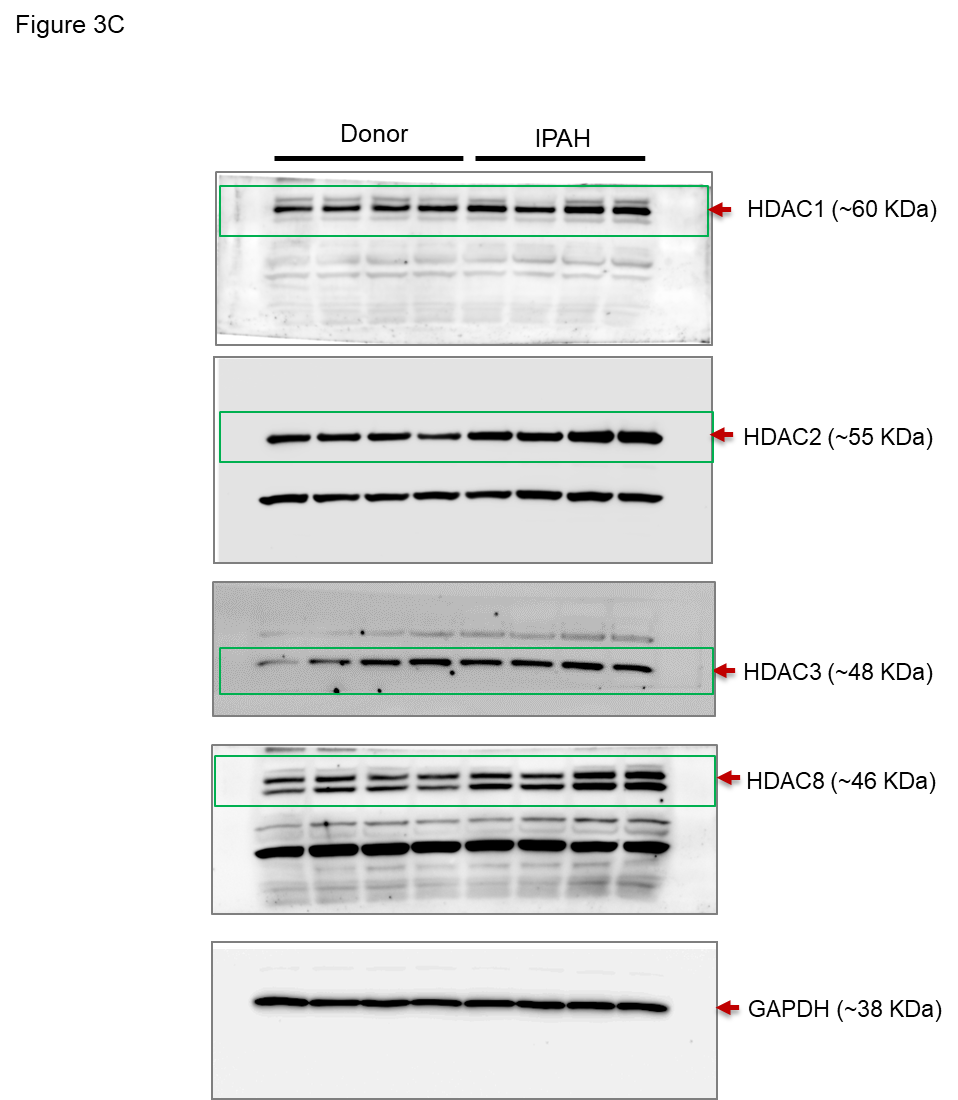
**

**
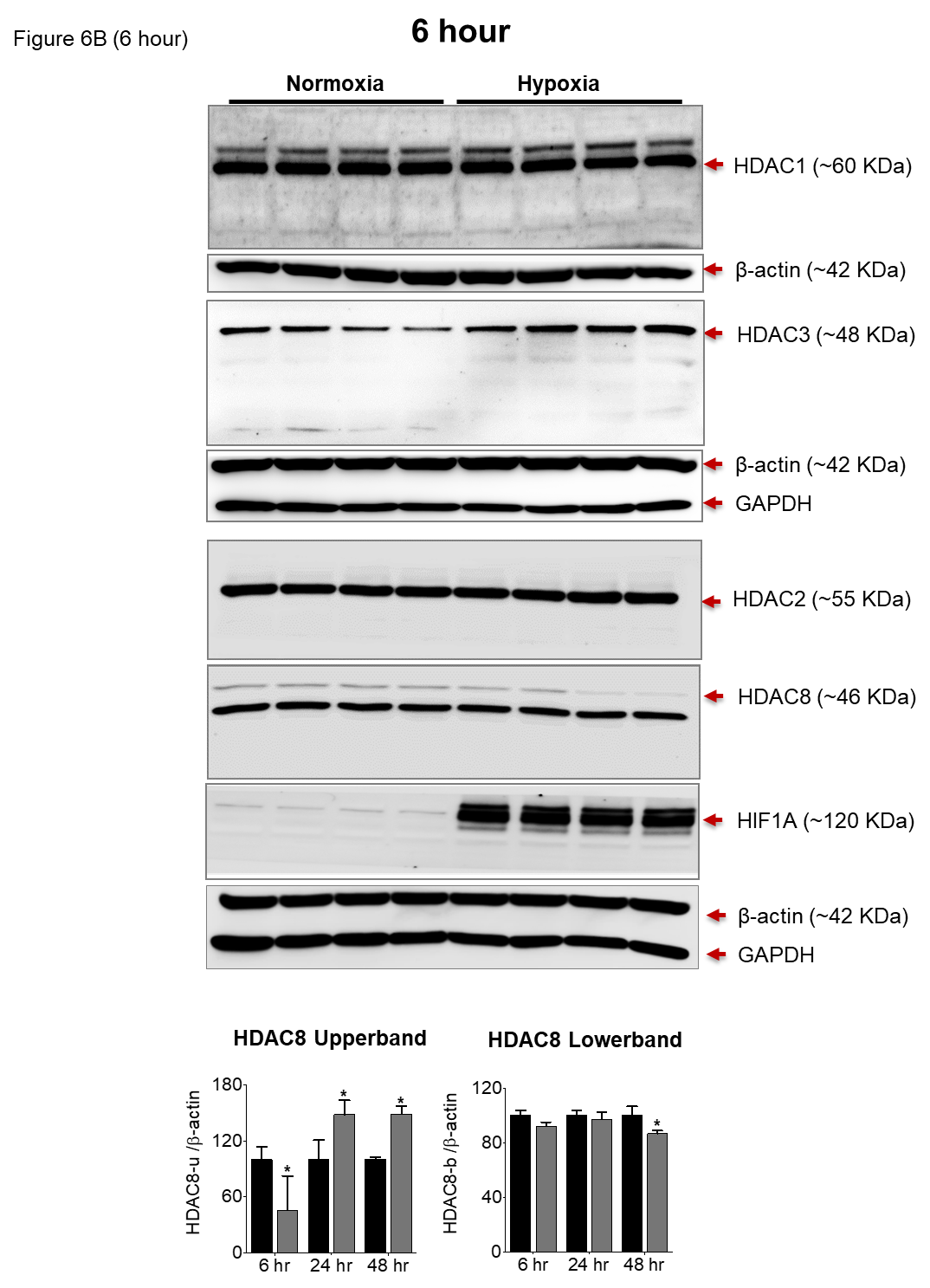
**

**
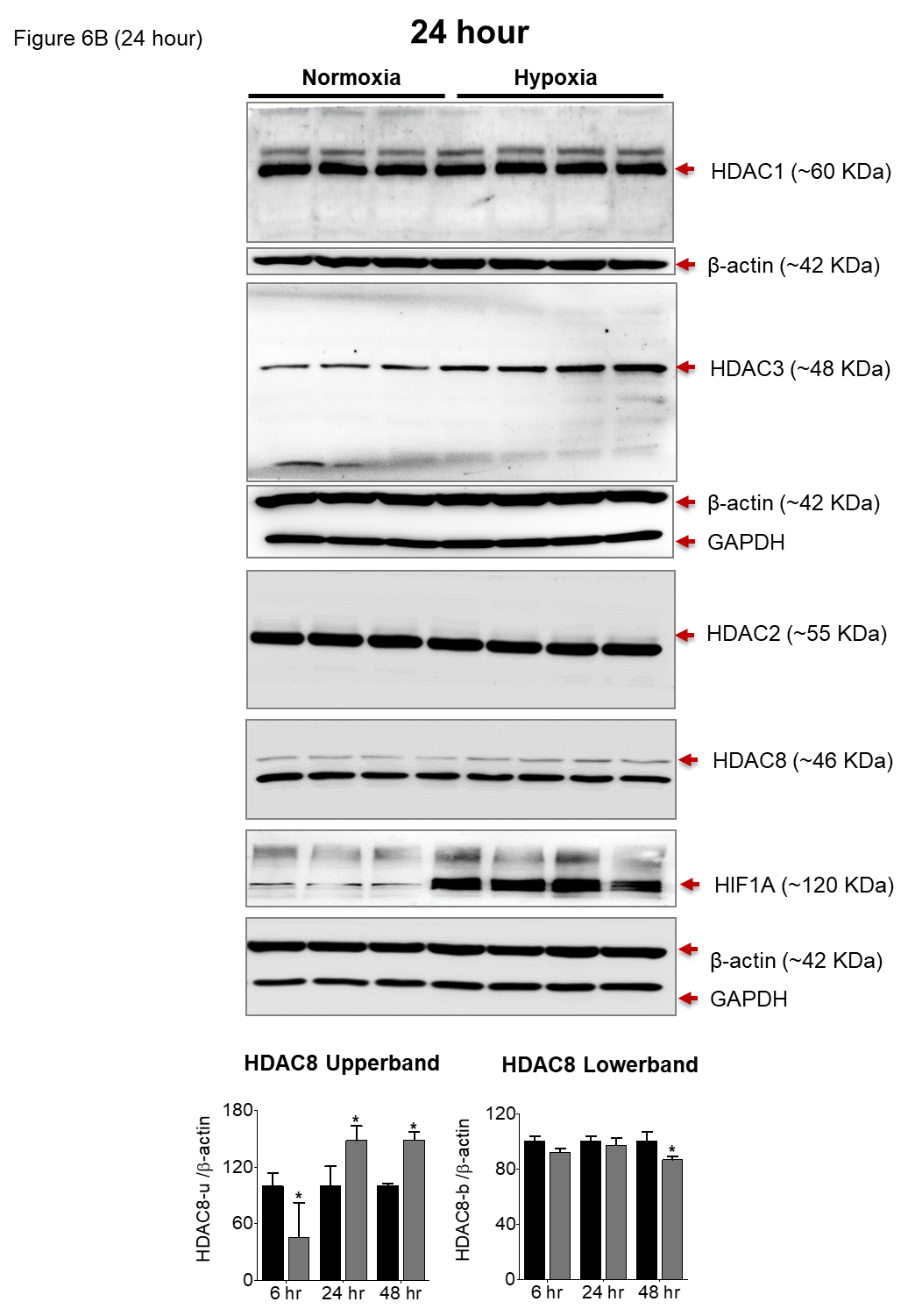
**

**
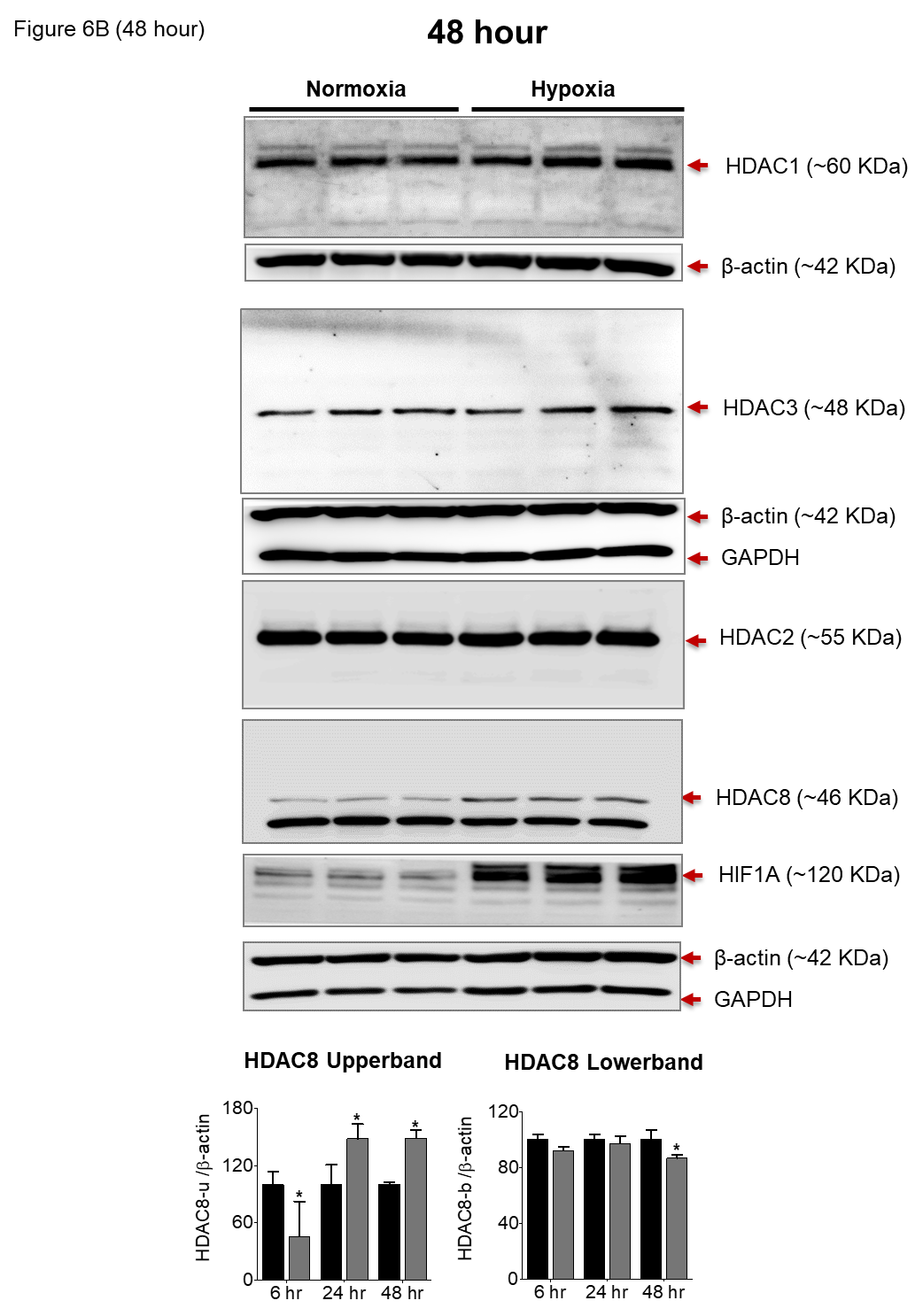
**

**
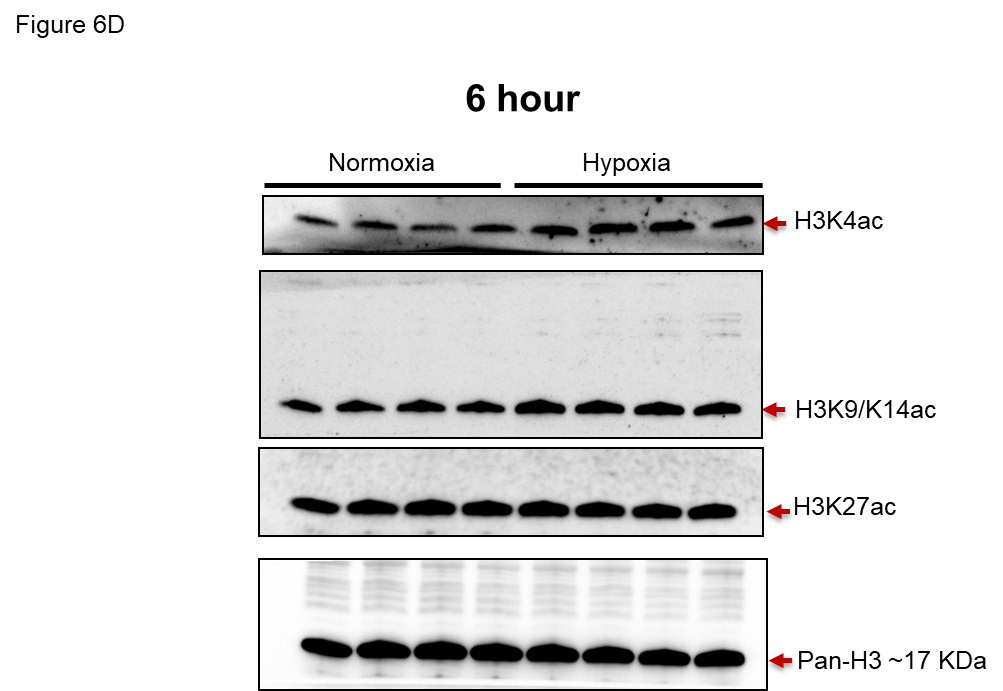
**

**
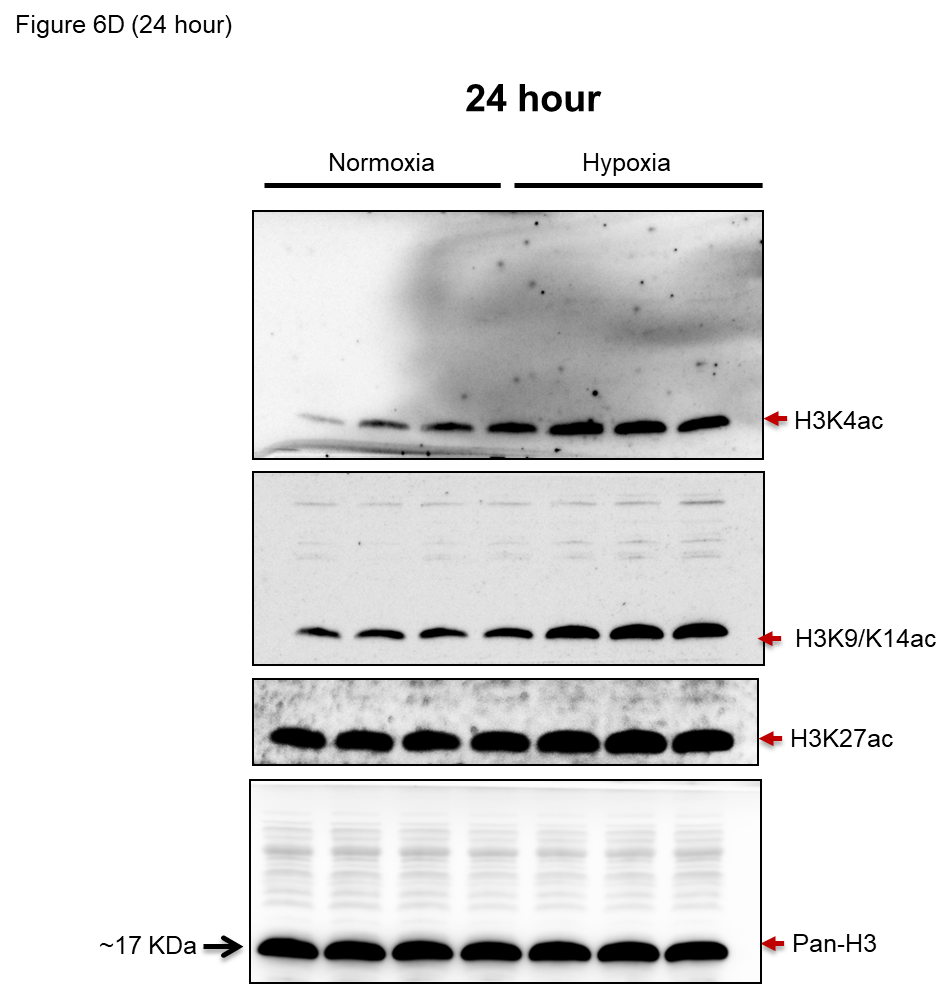
**

**
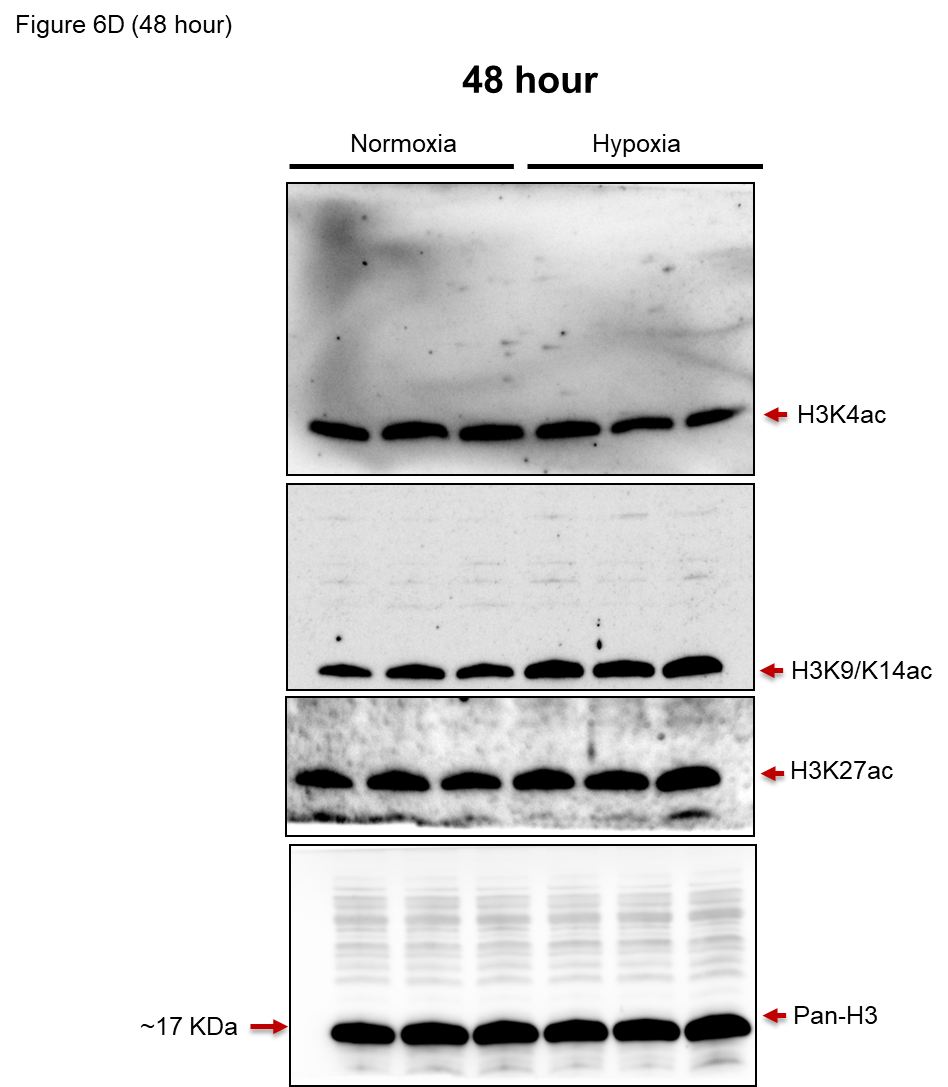
**

**
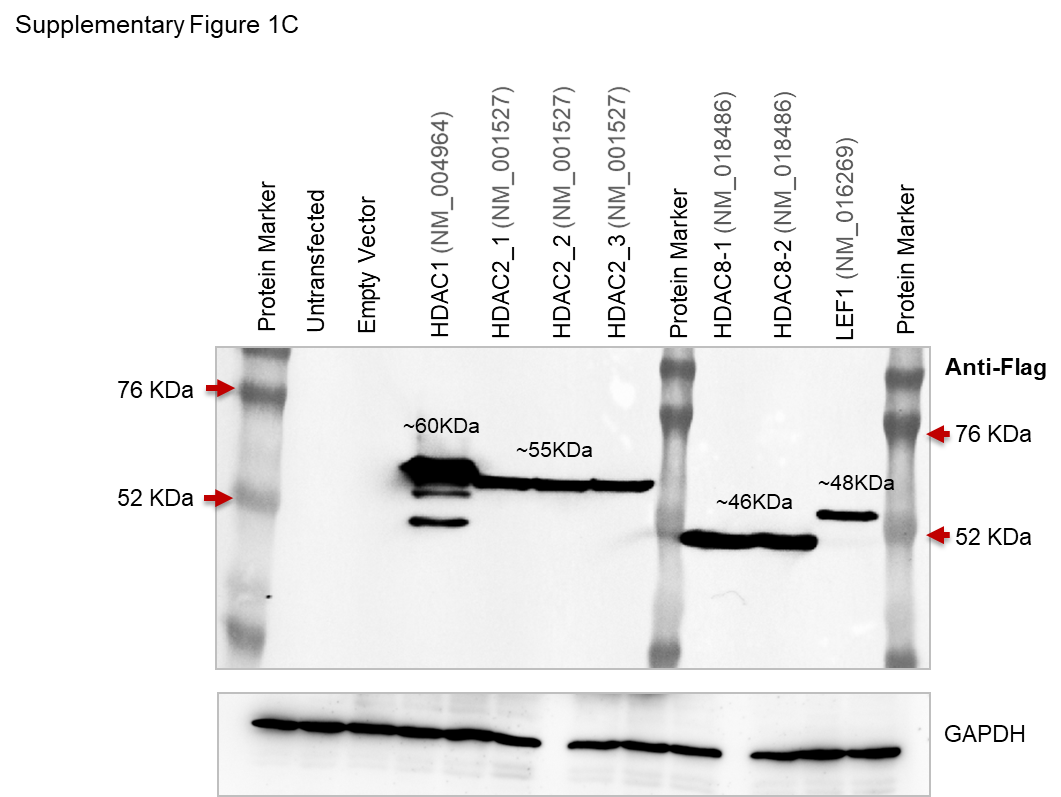
**

**
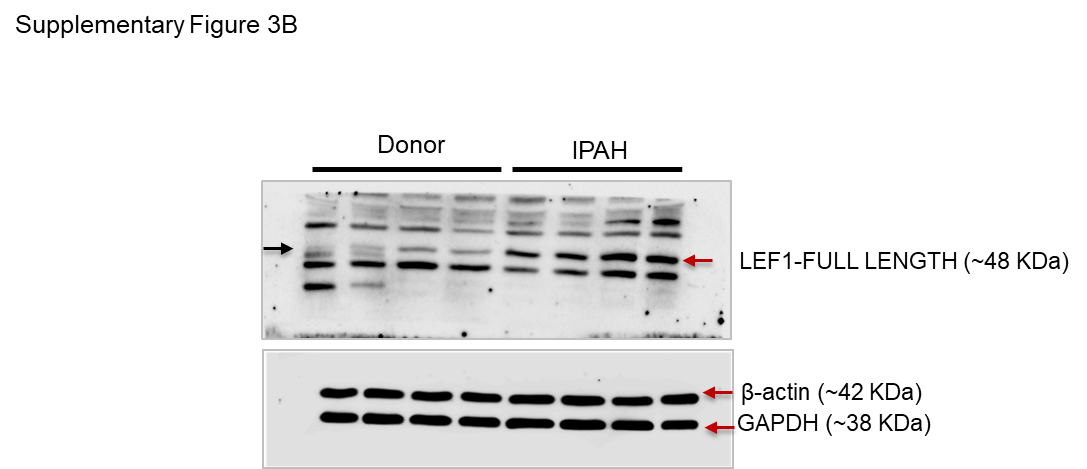
**
